# Arabidopsis DXO1 activates RNMT1 to methylate the mRNA guanosine cap

**DOI:** 10.1038/s41467-023-35903-8

**Published:** 2023-01-13

**Authors:** Chen Xiao, Kaien Li, Jingmin Hua, Zhao He, Feng Zhang, Qiongfang Li, Hailei Zhang, Lei Yang, Shuying Pan, Zongwei Cai, Zhiling Yu, Kam-Bo Wong, Yiji Xia

**Affiliations:** 1 Department of Biology, Hong Kong Baptist University, Hong Kong SAR, China; 2 State Key Laboratory of Environmental and Biological Analysis, Hong Kong Baptist University, Hong Kong SAR, China; 3 State Key Laboratory of Agrobiotechnology, School of Life Sciences, The Chinese University of Hong Kong, Hong Kong SAR, China; 4 School of Chinese Medicine, Hong Kong Baptist University, Hong Kong SAR, China

**Keywords:** Plant molecular biology, RNA metabolism, Plant development

## Abstract

Eukaryotic messenger RNA (mRNA) typically contains a methylated guanosine (m^7^G) cap, which mediates major steps of mRNA metabolism. Recently, some RNAs in both prokaryotic and eukaryotic organisms have been found to carry a non-canonical cap such as the NAD cap. Here we report that Arabidopsis DXO family protein AtDXO1, which was previously known to be a decapping enzyme for NAD-capped RNAs (NAD-RNA), is an essential component for m^7^G capping. AtDXO1 associates with and activates RNA guanosine-7 methyltransferase (AtRNMT1) to catalyze conversion of the guanosine cap to the m^7^G cap. *AtRNMT1* is an essential gene. Partial loss-of-function mutations of *AtRNMT1* and knockout mutation of *AtDXO1* reduce m^7^G-capped mRNA but increase G-capped mRNAs, leading to similar pleiotropic phenotypes, whereas overexpression of *AtRNMT1* partially restores the *atdxo1* phenotypes. This work reveals an important mechanism in m^7^G capping in plants by which the NAD-RNA decapping enzyme AtDXO1 is required for efficient guanosine cap methylation.

## Abstract (web summary)

Arabidopsis DXO1 is a member of the eukaryotic DXO family of decapping enzymes for NAD-capped RNAs. Here the authors show that DXO1 is an essential component in canonical m^7^G capping of mRNA and activates RNMT1 which methylates the guanosine cap to form the m^7^G cap.

## Introduction

In eukaryotic cells, a messenger RNA (mRNA) typically contains a methylated guanosine cap (m^7^G cap) that is added to its 5′ terminus through an unusual 5′−5′ triphosphate linkage^1–3^. Once the cap is added to the nascent transcript, it recruits proteins to form a cap-binding complex (CBC), which interacts with other proteins to mediate pre-mRNA processing, nuclear export, and translational initiation^2,4^. The cap also protects mRNAs from degradation by 5′ to 3′ exonucleases.

A nascent transcript initially begins with a 5′-triphosphate (5′-ppp) from the first transcribed nucleotide. The capping process occurs after transcription of 20–30 nucleotides^2,4^. First, 5′-ppp is hydrolyzed by an RNA 5′-triphosphatase to produce 5′-pp-RNA, which is then ‘capped’ by addition of a GMP by RNA guanyltransferase^5^. The resulting G cap is converted to the m^7^G cap by methylation at its N-7 position by an RNA guanosine-7 methyltransferase (RNMT). In mammals, the triphosphatase and guanyltransferase activities are present in a single polypeptide, which is also termed the capping enzyme (CE), whereas RNMT is encoded by a separate gene^5^. Mammalian RNMT has a basal activity that is increased several folds by association with a small (118 amino acid) protein, RNMT-Activating Miniprotein (RAM)^6^. These enzymes are also present in the cytoplasm of mammalian cells, where they can catalyze re-capping of decapped mRNAs, expanding the diversity of the mRNA population^7–10^.

It was once presumed that m^7^G capping is a constitutive process. However, recent discoveries show that RNA capping is a dynamic process that is mediated by developmental and environmental signals^8,11–14^. In yeast, cap methylation is regulated to temper translation in response to nutrient deficiency^14^. In mammals, G-cap methylation is regulated in a cell- and gene-specific manner^11–13^. The mammalian CE and RNMT are found in distinct complexes, allowing differential regulation of mRNA capping and cap methylation for specific transcripts^11,12^. Many transcription factors, such as c-Myc, are believed to upregulate cap methylation in addition to inducing transcription of their target genes^11,15^. Therefore, mRNA cap methylation serves as an important step in gene regulation. However, in plants, there is scant information on the m^7^G capping process and its roles in regulating gene expression. In recent years, non-canonical RNA caps, such as the NAD cap, have been found in some RNAs of both prokaryotes and eukaryotes, including *Arabidopsis thaliana*^16–24^, indicating more complexity than previously recognized in gene regulation through RNA capping and decapping.

Proteins of the DXO1 family in yeast and mammals are mostly known to hydrolyze the un-methylated and methylated G cap, to possess 5′ to 3′ exonuclease activity, and to play a role in monitoring quality of the RNA capping process^14,25^. Recently, mammalian and yeast DXO proteins were found to hydrolyze the NAD cap (deNADding) of NAD-capped RNAs (NAD-RNAs), and the mutation of mammalian DXO was reported to increase the level of NAD-capped RNAs^16^. Like mammals, the Arabidopsis genome encodes a single DXO protein (AtDXO1). AtDXO1 also possesses the deNADing activity and a weak 5′ to 3′ exonuclease activity, but is not active in hydrolyzing the m^7^G or G cap^26,27^. The knockout mutation of AtDXO1 causes pleiotropic phenotypes, including severe growth and developmental defects, low fertility, pale leaves, insensitivity to ABA, and extensive alteration in transcriptome profiles^24,26,27^. However, we and others have found that most of these mutant phenotypes can be fully complemented by a catalytically inactive AtDXO1, indicating that AtDXO1 has another important function unrelated to its enzymatic activity^26,27^.

In this report, we show that AtDXO1 is a critical component in m^7^G capping. AtDXO1 interacts with and activates AtRNMT1, which catalyzes conversion of the G-cap to the m^7^G cap. AtRNMT1 has a basal cap methylation activity that is increased in the presence of AtDXO1. The *atrnmt1-1* mutation leads to early embryonic lethality. The *rnmt1-2* and *rnmt1-3* mutations (two weaker alleles) and the *atdxo1* mutation all reduce the proportion of m^7^G-capped cellular mRNAs while increasing G-capped mRNAs, leading to similar pleiotropic phenotypes. Overexpression of *AtRNMT1* partially restores the *atdxo1* phenotypes. The defect in cap methylation caused by these mutations affects transcript levels of overlapping sets of genes. Our study reveals a novel mechanism of RNA cap methylation and raises a possibility that NAD decapping and m^7^G capping might be connected through the DXO family protein.

## Results

### The plant-specific N-terminal extension of DXO1 interacts with RNMT1

Previous studies indicate that AtDXO1 (abbreviated as DXO1 hereafter) has another important function in addition to its enzymatic function. To gain information on DXO1 function, we carried out a yeast two-hybrid (Y2H) screen to identify potential interacting proteins. The full-length DXO1 fused to the DNA binding domain (BD-DXO1) was used as bait to screen the Mate & Plate™ Universal Arabidopsis library (Takara). Activation domain (AD)-containing plasmids were isolated from the colonies that grew on the selective medium and sequenced to identify the cloned Arabidopsis cDNA sequences fused with the AD. Two of the clones were found to contain At3g20650 fused in-frame with the AD. At3g20650 was previously named cap methyltransferase (CMT) based on its sequence similarity to known RNMTs in yeast and animals^28^. We re-named At3g20650 to AtRNMT1 in this report, because CMT has been used as a symbol for CHROMOMETHYLASE, which methylates DNA.

**Fig. 1:**
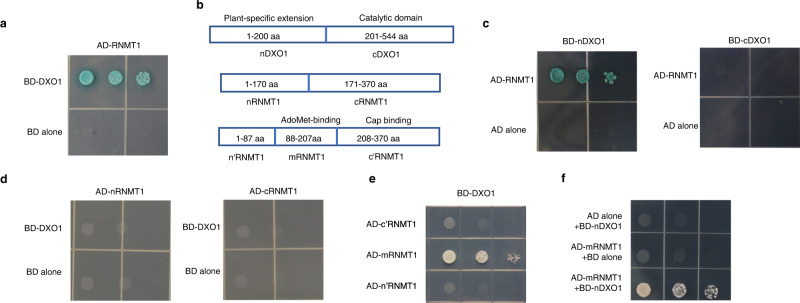
DXO1 interacts with RNMT1 in the yeast two-hybrid system. **a** Yeast cells transformed by *pGADT7-RNMT* (AD-RNMT1) together with *pGBKT7-DXO1* (BD-DXO1), but not with the empty vector *pGBDT7* (BD alone), were able to grow on the selective medium. **b** Schematic diagrams of different regions of DXO1 and RNMT1 tested for their interaction by Y2H assay and other assays in this report. **c** The plant-specific N-terminal extension of DXO1 (nDXO1) interacted with RNMT1. Yeast cells transformed by *BD-RNMT1* together with *BD-nDXO1*, but not with *BD-cDXO1*, were able to grow on the selective medium. **d** nRNMT1 and cRNMT1 did not interact with DXO1. Yeast cells transformed by *BD-DXO1* together with *AD-nRNMT1* or *AD-cRNMT1* could not grow on the selective medium. **e** The middle region of RNMT1 (88-207 aa; mRNMT1) interacted with DXO1. Yeast cells transformed by *BD-DXO1* together with *AD-n’RNMT, AD-mRNMT*, or *AD-c’RNMT* were cultured on the selective medium. **f** mRNMT1 interacted with nDXO1. Yeast cells transformed by *BD-nDXO1* together with *AD-mRNMT1* were able to grow on the selective medium. Yeast cells were grown on the selective medium (SD medium without Leu, Trp, His and Ade and supplemented with/without X-α-Gal and AbA) for 3 days at 30 °C.

The interaction between DXO1 and AtRNMT1 (abbreviates as RNMT1 hereafter) was further confirmed using the Y2H assay (Fig. 1a). DXO1 has a plant-specific N-terminal extension of about 200 amino acids (aa) in addition to the conserved DXO catalytic domain (Fig. 1b)^26,27^. The first 120 aa of this N-terminal extension is predicted to be intrinsically unstructured regions that are known to interact with proteins and other biomolecules^29^, whereas the C-terminus of DXO1 is predicted to have RNA-binding region(s) (Supplementary Fig. 1). To determine which regions of DXO1 and RNMT1 are involved in their interaction, we divided DXO1 into nDXO1 (the 1-200 aa plant-specific extension) and cDXO1 (Fig. 1b). In Y2H, RNMT1 interacted with nDXO1 but not with cDXO1 (Fig. 1c). We also split RNMT1 into nRNMT1 (1–170aa) and cRNMT1 (171–370aa) (Fig. 1b and Supplementary Fig. 2). Neither nRNMT1 nor cRNMT1 alone interacted with DXO1 (Fig. 1d), suggesting that the interaction domain of RNMT1 spans these two regions. We then divided RNMT1 into three regions: 1-87 aa (n’RNMT1) which is less conserved, 88-207aa (mRNMT1, the middle region) which contains the highly conserved S-adenosylmethionine (AdoMet)-binding motif VLxI*/*LxxGxGxDL, and 208-370aa (c’RNMT1) which contains several conserved residues for cap binding of known RNMTs^30^ (Fig. 1b and Supplementary Fig. 2). The result showed that the middle region of RNMT1 interacts with DXO1 (Fig. 1e). Another Y2H analysis further showed that the DXO1/RNMT1 interaction occurred between the nDXO1 region and the mRNMT1 region (Fig. 1f).

**Fig. 2:**
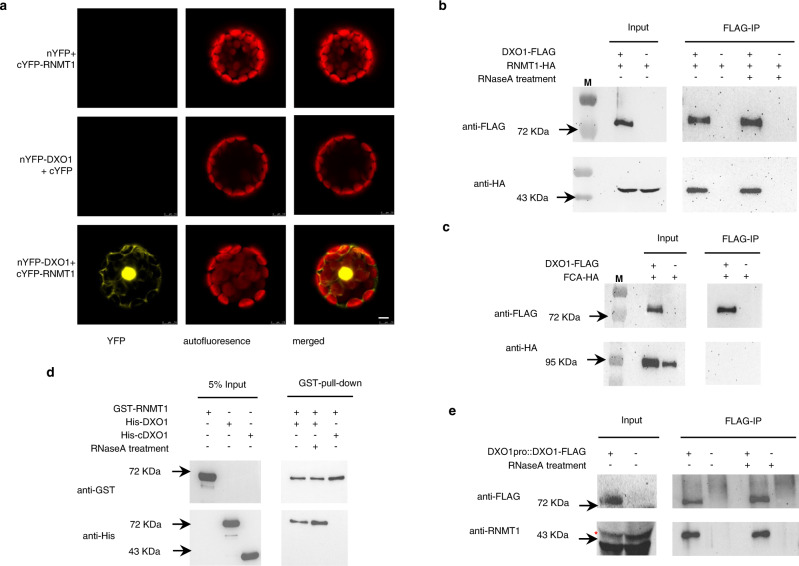
DXO1 and RNMT1 interact *in planta*. **a** BiFC assay. The pairs of *nYFP* and *cYFP-RNMT1, nYFP-DXO1* and *cYFP*, or *nYFP-DXO1* and *cYFP-RNMT1* were co-expressed in Arabidopsis protoplasts. *nYFP* and *cYFP* are the empty vectors used as negative controls. The YFP signal was detected in the nucleus and the cytosol only when *nYFP-DXO1* and *cYFP-RNMT1* were co-expressed. Bar: 5 μM. **b** Co-immunoprecipitation assay showed DXO1 and RNMT1 interacted in Arabidopsis protoplasts. The *RNMT1-HA* fusion gene under the control of the *35S* promoter was transfected into protoplasts of the *35S:DXO1-FLAG* transgenic plants and WT plants. Proteins extracted from the protoplasts with or without RNaseA treatment were immunoprecipitated using the anti-FLAG M2 resin. The immuno-precipitates were examined by Western blotting using the anti-FLAG or anti-HA antibodies. RNMT1-HA was detected in the immuno-precipitate when it was expressed in the *35S:DXO1:FLAG* cells. M: protein molecular weight markers. **c** FCA-HA (used as a control) did not interact with DXO1-FLAG. The experiment was carried out in the same way as in **b** except that the *35Spro::FCA-HA* fusion construct was transfected into protoplasts of the *35S:DXO1-FLAG* transgenic and WT plants. **d** In vitro GST-pull-down assay. Purified GST-RNMT1 was mixed with His-DXO1 or His-cDXO1. Proteins were precipitated by glutathione agarose beads. Anti-GST and anti-His antibodies were used to detect GST-RNMT1 and His-DXO1 or His-cDXO1, respectively, in the precipitates by Western blotting. His-DXO1, but not His-cDXO1, was found to be co-immunoprecipitated. **e** Co-IP assay in the stable transgenic plants. Proteins extracted from 12-day-old seedlings of the transgenic line carrying *DXO1pro::DXO1-FLAG* and WT (with or without RNaseA treatment) was immunoprecipitated using the anti-FLAG M2 resin. DXO1-FLAG and RNMT1 in the precipitates were detected by the anti-FLAG and anti-RNMT1 antibodies, respectively, by Western blotting. The asterisk indicates the RNMT1 protein. The arrows in **b**–**e** point to the positions of the protein molecular weight markers. **a**–**e** Represent the results from one of three independently performed experiments with similar results.

To determine if DXO1 and RNMT1 interact *in planta*, we first used bimolecular fluorescence complement (BiFC) assay. When *nYFP-DXO1* and *cYFP-RNMT1* fusion proteins were transiently expressed in Arabidopsis protoplasts, the YFP signal was detected strongly in the nuclei and weakly in the cytosol (Fig. 2a). We have previously found that DXO1 is localized mostly in the nucleus, but some in the cytosol^26^, while RNMT1 also shows a similar subcellular localization pattern (see below). We then carried out co-immunoprecipitation (co-IP) assays. RNMT1 fused with the HA tag was expressed in protoplasts of either a transgenic line carrying a *35S promoter:DXO1-FLAG* fusion gene or WT plants. Proteins were immunoprecipitated by the anti-FLAG antibodies to pull-down DXO1-FLAG fusion proteins. By probing the immunoprecipitates with anti-HA antibodies in Western blots, it showed that RNMT1-HA fusion protein was co-precipitated by the anti-FLAG antibodies (Fig. 2b).

There exists a possibility that RNMT1 and DXO1 might interact indirectly by tethering to the same RNA molecule. To test that, extracted protein samples were treated with RNase A to degrade any associated RNAs before the co-IP assay. A known Arabidopsis RNA-binding protein, FCA^31^, was used as a negative control. It was found that the DXO1/RNMT1 interaction was not affected by the RNase A treatment (Fig. 2b) whereas FCA did not interact with DXO1 (Fig. 2c). Furthermore, GST-tagged RNMT1, His-tagged DXO1, and His-tagged cDXO1 proteins were expressed in and purified from *E. coli* and used in an in vitro GST-pull-down assay in the presence of RNase A. The result also supports that RNMT1 and DXO1 interacted directly but cDXO1 did not interact with RNMT1 (Fig. 2d). In addition to the co-IP assay using protoplasts, proteins were extracted from the stable Arabidopsis transgenic lines expressing the *DXO1pro::DXO1-FLAG* transgene and treated with RNase A. FLAG-DXO1 were pulled-down by the anti-FLAG antibodies. RNMT1 from the native *RNMT1* gene (detected by the anti-RNMT1 polyclonal antibodies) was found to be co-immunoprecipitated by the anti-FLAG antibodies (Fig. 2e). Together, the above results reveal that DXO1 directly interacts with RNMT1.

### DXO1 enhances RNMT1’s RNA cap methyl-transferase activity

Supplementary Fig. 2 shows a multiple sequence alignment between Arabidopsis RNMT1, its closest Arabidopsis homolog (At3g52210), a rice homolog, and the known RNMTs^32^ from humans and yeast. The yeast and mammalian RNMTs consist of 430–480 amino acids (aa), are highly conserved in the 120–350-aa middle region, C-terminal region containing the conserved catalytic domain, and share a low sequence similarity in their ~120-aa N-termini^32^.

Arabidopsis RNMT1 consists of 370 amino acids and does not have a long, non-catalytic N-terminal domain like human RNMT1. Its ~40-aa N-terminal and 30-aa C-terminal regions are unique, while the remaining 300-aa central region shares approximately 40% identity to the catalytic domain of the yeast and human RNMTs. Arabidopsis RNMT1 shares about 25% overall sequence identity with its closest Arabidopsis homolog, AT3G52210, and a much lower sequence identify with other methyltransferase family proteins encoded by the Arabidopsis genome. At3g52210 shares ~19% sequence identity to the catalytic domains of yeast and human RNMTs and is unlikely to have an RNMT-like activity as its lacks the highly conserved AdoMet binding motif VLxI*/*LxxGxGxDL and some other highly conserved residues (Supplementary Fig. 2) critical for the RNA cap methyltransferase activity^30,32–34^. Therefore, RNMT1 is likely the sole cap methyltransferase in Arabidopsis.

**Fig. 3:**
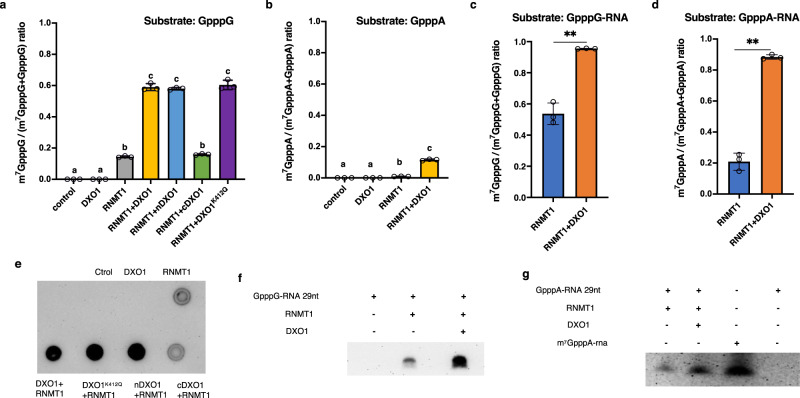
RNMT1 is an RNA cap methyl transferase and activated by DXO1. Conversion of GpppG (**a**), GpppA (**b**), GpppG-RNA (29-nt (**c**), and GpppA-RNA (29-nt (**d**), to m^7^GpppG, m^7^GpppA, m^7^GpppG-RNA, and m^7^GpppA-RNA, respectively, by RNMT1 alone or with DXO1, nDXO1, cDXO1, and DXO1^K412Q^. Control: the reaction with no protein. m^7^GpppA-RNA and m^7^GpppG-RNA were digested by nuclease P1 to release GpppA, GpppG, m7GpppA, and m7GpppG, which were detected and quantified by mass spectrometry. Different letters indicate significant difference by one-way ANOVA, Tukey’s test (*p* < 0.05) (**a**, **b**). ** indicates *p* < *0.01* determined by two-tailed unpaired *t*-test (**c**, **d**). Data in **a**–**d** are mean ± SD (*n* = 3 biologically independent samples). Conversion of GpppG to m^7^GpppG, GpppG-RNA to m^7^GpppG-RNA, and GpppA-RNA to m^7^GpppA-RNA by RNMT1 alone or with DXO1 were determined by dot blotting (**e**) and RNA blotting (for m^7^GpppG-RNA and m^7^GpppA-RNA) (**f**, **g**). The synthetic m^7^GpppA-RNA, which has the same sequence as GpppA-RNA was used as a positive control in RNA blotting analysis. The blots were probed by the anti-m^7^G antibodies.

We carried out in vitro enzymatic assays to experimentally determine if Arabidopsis RNMT1 is indeed an RNA guanosine cap methyltransferase. Recombinant RNMT1 was expressed in and purified from *E. coli*. DXO1, nDXO1, cDXO1, and the catalytically inactive DXO1^K412Q^ were similarly prepared and included in the assay. The K412Q mutation was previously reported to abolish the DXO1’s enzymatic activity^26^. The methyltransferase activity of RNMT1 was first determined using the unmethylated RNA cap analogs GpppA or GpppG as substrates and AdoMet as the methyl donor. After the reactions, the products were analyzed by mass spectrometry to determine and quantify conversion of GpppA and GpppG to m^7^GpppA and m^7^GpppG, respectively. When GpppG was used as the substrate, approximately 15% was converted to m^7^GpppG by RNMT1, whereas DXO1 was not found to possess such an activity (Fig. 3a). However, RNMT1 only showed a very weak activity on GpppA (Fig. 3b). As the dinucleotides GpppA and GpppG are not real RNA substrates for cap methytransferases, we synthesized a 29-nucleotide G-capped RNA with adenosine or guanine as the first transcribed nucleotide (GpppA-RNA and GpppG-RNA) (see the Methods) and used them as the substrates. After the reactions, the samples were digested with nuclease P1 to release the cap linked with the first nucleotide (i.e., GpppA/G or m^7^GpppA/G) from the other nucleotides, and GpppA/G and m^7^GpppA/G were quantified by mass spectrometry. Approximately 20% of GpppA-RNA and 50% of GpppG-RNA were converted to m^7^G-capped RNAs by RNMT1 (Fig. 3c, d).

To determine if the interaction with DXO1 has any effect on RNMT1 activity, DXO1 was added together with RNMT1 in the enzymatic assay. On the substrate GpppG, the presence of DXO1 increased RNMT1 activity by several folds (Fig. 3a and Supplementary Fig. 3a). The presence of nDXO1 and DXO1^K412Q^, but not cDXO1, similarly enhanced the RNMT1’s activity (Fig. 3a and Supplementary Fig. 3a), indicating that the plant-specific N-terminal extension is essential for enhancing RNMT1 activity. The presence of DXO1 also significantly increased RNMT1 activity on GpppA (Fig. 3b and Supplementary Fig. 3b). In addition, DXO1 significantly enhanced the RNMT1 activity when GpppA-RNA and GpppG-RNA were used as the substrate: nearly 90% of both G-capped RNA substrates were converted into the m^7^G-capped products (Fig. 3c, d and Supplementary Fig. 3c, d).

In addition to the enzymatic assay of RNMT1 activity by the quantification using mass spectrometry, we used the anti-m^7^G antibodies to detect the methylated cap on the reaction products using dot blotting and Northern blotting for semi-quantitative assay (Figs. 3e–g). Consistent with the mass spectrometry analysis, the blotting analysis also showed that RNMT1 possessed a basal activity of RNA cap methyltransferase and that DXO1 served as its activator.

### RNMT1 is constitutively expressed

Because of the importance of the m^7^G cap for mRNA metabolism and function, *RNMT1* is expected to be expressed in all living cells. Indeed, the data from the publicly available transcriptome databases (GENEVESTIGATOR and Arabidopsis eFP Browser) show that *RNMT1* was expressed in all tissues at different developmental stages, although its transcript levels could differ by a few folds in different organs (Supplementary Fig. 4a). Using reverse transcription followed by quantitative PCR (qPCR), the *RNMT1* transcripts were detected in all organs we examined, and its levels differed by approximately two folds (Supplementary Fig. 4b).

To determine the subcellular localization of RNMT1, the *RNMT1* genomic sequence was fused in frame with eGFP and transiently expressed under the control of the cauliflower mosaic virus (CaMV) 35S promoter (*35S:RNMT1-eGFP*) in Arabidopsis protoplasts. A strong GFP signal was found localized in the nucleus, whereas a weak GFP signal was also detected in the cytosol (Supplementary Fig. 5). The *RNMT1-eGFP* transgene was capable of complementing the *rnmt1* mutant phenotype (see below), indicating that the fusion gene functions normally.

### *RNMT1* is an essential gene

We obtained an Arabidopsis line (SAIL_1252_D05) from the Nottingham Arabidopsis Stock Center (NASC) (http://Arabidopsis.info/) containing a T-DNA insertion in the eleventh exon of *RMNT1* (named *rnmt1-1*; abbreviated as *rnmt1*) (Supplementary Fig. 6a). We were able to obtain *rnmt1/+* heterozygous plants but no homozygous insertion line was recovered among over 200 examined progenies from self-pollinated *rnmt1/+* heterozygous plants, indicating that the homozygous *rnmt1* mutation is lethal. The progenies from self-pollinated *rnmt1/+* plants showed a 1.91:1 (113:57) segregation ratio of the *rnmt1/+*:+/+ genotypes based on the genotyping analysis by PCR, which fits the 2:1 segregation ratio by the Chi-square test. The *rnmt1/+* plants exhibited a similar phenotype as WT (Supplementary Fig. 6b), except that 24% (213/885) of the developing seeds in the siliques of *rnmt1/+* plants were abnormal which fits the 3:1 segregation ratio by the Chi-square test, whereas nearly all seeds (697/700) from WT were normal. These abnormal seeds were pale at the early stage of seed development and later turned brownish and shrunken (Supplementary Fig. 6c).

The above results indicated that *RNMT1* is essential for seed development. We transformed the *RNMT1pro:RNMT1-eGFP* transgene into *rnmt1/+* plants. From the progeny, we obtained plants homozygous for the *rnmt1* allele that carried the transgene. These transgenic lines showed no discernible phenotype from WT (Supplementary Fig. 6d). The result demonstrated that the seed abortion phenotype associated with the heterozygous plants was indeed caused by disruption of the *RNMT1* gene by the T-DNA insertion.

### The *rnmt1* mutation causes early embryonic lethality

Genotyping of the progenies from reciprocal crosses between the *rnmt1/+* and WT plants showed that the progenies segregated 1:1 (+/+:*rnmt1*/+) (Supplementary Table 1), indicating that the *rnmt1* allele was transmitted equally well as the WT allele through both male and female gametes gametophytes. The segregation results were supported by microscopic observation. Pollen grains from the *rnmt1/+* mutant and WT all showed nearly 100% viability (Supplementary Fig. 7a). Furthermore, pollen grains from *rnmt1/+* plants did not show abnormality when examined by scanning electron microscopy (Supplementary Fig. 7b). Similarly, morphological observation by scanning electron microscopy did not find any obvious morphological difference in ovaries from WT and *rnmt1/+* plants (Supplementary Fig. 7c).

The above results revealed that the *rnmt1* mutation does not affect male or female gametogenesis, but homozygous *rnmt1* zygotes could not develop into normal seeds. To determine which stages of embryogenesis are affected by the *rnmt1* mutation, developing embryos in the *rnmt1/+* plants and WT plants were observed via the whole mount clearing technique^35^. During normal embryogenesis, a zygote undergoes several cycles of cell division to enter the globular stage and then proceeds to the heart, torpedo, and curled-cotyledon stages (Supplementary Fig. 8a). In the self-fertilized *rnmt1/+* siliques, we did not find any obvious morphological difference in developing embryos during the globular stage. However, as time progressed, abnormal embryos from the *rnmt1/+* plants were all arrested at the globular stage (Supplementary Fig. 8a, b).

### *rnmt1-2* and *rnmt1-3* share similar phenotypes to those of *dxo1*

**Fig. 4:**
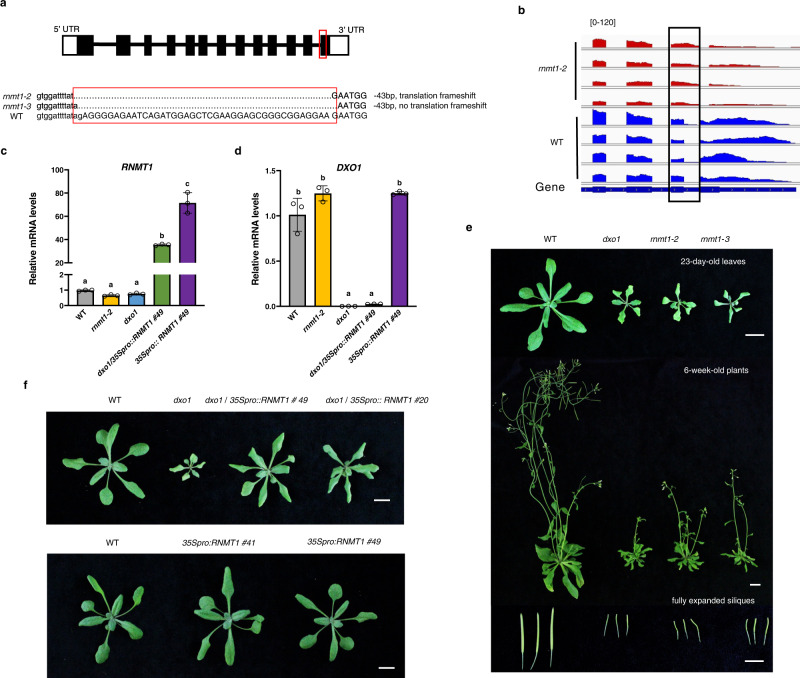
The *rnmt1-2* and *rnmt1-3* mutations lead to phenotypes similar to those of *dxo1*. **a** Schematic diagram of the *rnmt1-2* and *rnmt1-3* alleles, both of which have a 43 bp deletion from one and two bases, respectively, before the last exon. The red frames indicate the deleted regions. The capital letters represent exon sequences and the lowercase represents intron sequences of *RNMT1*. **b** Integrative genomic viewer (IGV) diagram of *RNMT1* gene splicing pattern in *rnmt1-2* and WT. The scale of each track is identical. The *Y*-axis ranges from 0 to 200. There are four repeats in each lines. Gene structure are shown at the bottom: exons (thick boxes), introns (lines) and 3′ UTR (lines). The black frame includes the last intron which was retained in the transcripts from the *rnmt1-2* allele. Transcript levels of *RNMT1* (**c**) and *DXO1* (**d**) in 12-day-old seedlings of WT, *rnmt1-2*, *dxo1*, *dxo1/35pro::RNMT1* and *RNMT1* overexpression lines relative to that in WT as determined by RT-qPCR. *UBQUITIN5* (*UBQ5*) was used as the internal control. Different letters indicate significant differences by one-way ANOVA, Tukey’s test (*p* < 0.05). Data in **c** and **d** are mean ± SD (*n* = 3 biologically independent samples). **e** Morphological phenotypes of WT, *dxo1, rnmt1-2*, and *rnmt1-3*. **f** Phenotypes of WT, *dxo1*, *dxo1/35Spro:RNMT1* and *35Spro::RNMT1* lines. RNMT1 overexpression partially complemented the *dxo1* phenotype. Bar: 1 cm (**e**, **f**).

The lethal phenotype caused by *rnmt1* prevented us from obtaining homozygous mutant plants for further functional characterization of *RNMT1*. To overcome this hurdle, we used the CRISPR/Cas9 system in aim of introducing mutation in the last (13th) exon of *RNMT1* to create a non-lethal weaker allele. We obtained two independent alleles, named *rnmt1-2* and *rnmt1-3* (Fig. 4a). Both alleles have a 43-bp deletion, starting from the last one and two bases of its last intron, respectively. In addition to the deletion, both mutations are expected to block splicing of the last intron, as confirmed based on Integrative Genomics Viewer (IGV) analysis of the *RNMT1* transcripts from RNA-sequencing data (see below) of WT and *rnmt1-2* (Fig. 4b). The proteins coded by these two mutant alleles are expected to miss last 30 aa coded by the last exon, which are not in the highly conserved catalytic domain (Supplementary Fig. 2). The *RNMT1* transcript level in *rnmt1-2* was also reduced by approximately 30% compared to WT (Fig. 4c). The homozygous *rnmt1-2* and *rnmt1-3* mutants are viable but exhibit pleotropic growth and developmental defects that resemble the phenotypes of *dxo1*, including the small statue with narrow pale leaves and high infertility (Fig. 4e). However, the *rnmt1-2* and *rnmt1-3* plants grew slightly bigger than *dxo1* and also bore more seeds.

### Overexpression of *RNMT1* partially complement the *dxo1* phenotypes

The similar phenotypes of *dxo1* and the weak *rnmt1* alleles as well as the finding of DXO1 as an activator of RNMT1 suggest that the phenotypes caused by the *dxo1* mutation could also be due to a defect in RNA cap methylation. We transformed the *dxo1* line with a construct in which the expression of *RNMT1* is under the control of the 35S promoter (*35Spro:RNMT1*). Overexpression of *RNMT1* partially complemented the *dxo1* phenotypes (Fig. 4f), indicating that overexpression of RNMT1 partially amends the defect in RNA cap methylation caused by loss of DXO1. Overexpression of RNMT1 in the WT background did not result in any discernible phenotype from WT plants (Fig. 4f). Loss of function or overexpression of *RNMT1* did not lead to obvious alternation of the *DXO1* transcript level (Fig. 4d).

### RNMT1 and DXO1 function in mRNA G-cap methylation in vivo

**Fig. 5:**
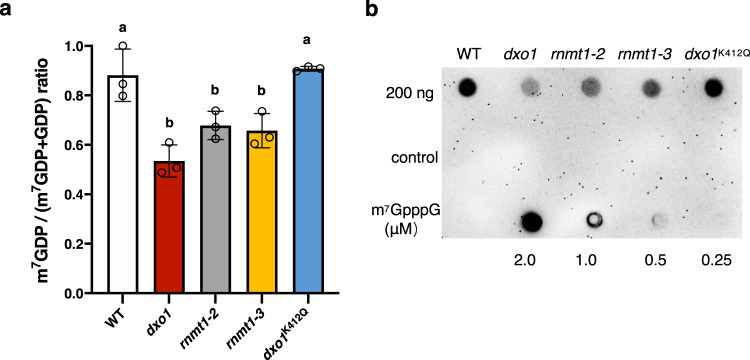
RNMT1 and DXO1 function in RNA cap methylation in vivo. **a** Quantification of G-capped and m^7^G-capped RNAs in WT, *rnmt1-2, rnmt1-3, dxo1*, and *dxo1/DXO1*^*K412Q*^ by mass spectrometry. Caps of poly(A)-enriched mRNAs (4 μg) from 4-week-old plants were hydrolyzed by the mRNA Decapping Enzyme to release m^7^GDP or GDP for mass spectrometry analysis. The mRNA samples without the enzyme treatment were used as a negative control. Different letters indicate significant differences by one-way ANOVA, Tukey’s test (*p* < 0.05). Data are mean ± SD (*n* = 3 biologically independent samples). **b** Dot blotting analysis for detection of m^7^G-mRNAs. The mRNA samples were spotted onto the nylon membrane and probed with the m^7^G antibodies. For the negative control, mRNAs were decapped with the mRNA Decapping Enzyme before being spotted onto the membrane. Different concentration of m^7^GpppG was spotted to the membrane as standards. **b** represents the results from one of three independently performed experiments with similar results.

The availability of the non-lethal *rnmt1* mutations and the *dxo1* mutation allowed us to determine if RNMT1 and DXO1 function in G cap methylation in plant cells. The poly(A)-enriched RNA samples from 4-week-old plants of WT, *rnmt1-2*, *rnmt1-3*, *dxo1*, and *dxo1*^K412Q^ were digested by the mRNA Decapping Enzyme, which hydrolyzes the pyrophosphate bond to release GDP from the G cap and m^7^GDP from the m^7^G cap. GDP and m^7^GDP were quantified using mass spectrometry. Compared to WT, the ratio of the m^7^G cap to total caps (G cap and m^7^G cap) was approximately 25% lower in *rnmt1-2*, *rnmt1-3*, and *dxo1*, whereas the G-capped mRNA levels were significantly increased in the mutants (Fig. 5a and Supplementary Fig. 9). There was no significant difference in the m^7^G and G cap content between WT and the *dxo1/DXO1*^*K412Q*^ plants (Fig. 5a and Supplementary Fig. 9). A similar result was obtained when the anti-m^7^G antibodies was used to detect m^7^G-RNAs in the RNA samples using RNA blot analysis (Fig. 5b).

### *rnmt1-2* and *dxo1* affect transcript levels of overlapped sets of genes

**Fig. 6:**
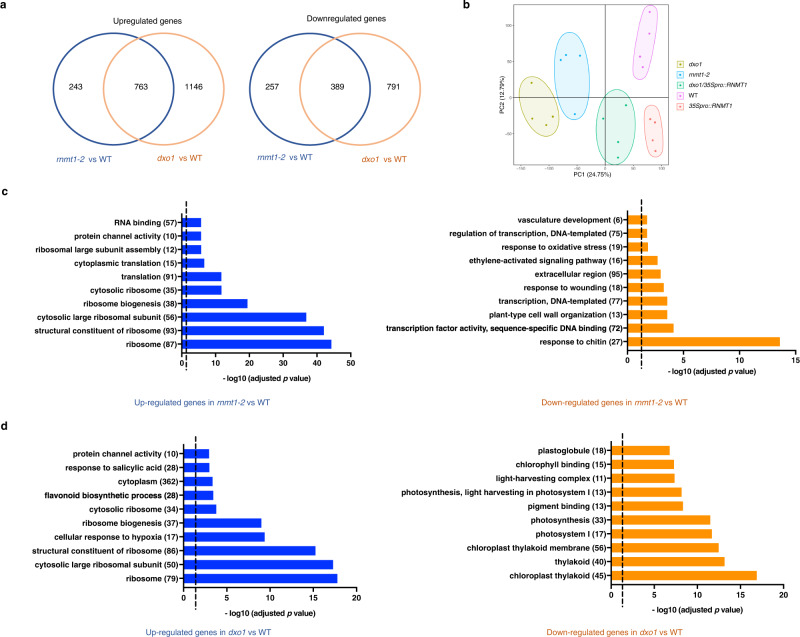
Differentially expressed genes caused by the *rnmt1-2* and *dxo1* mutations. **a** Venn diagrams show the number of up- and downregulated DEGs (in relation to expression in WT) that overlap between the *rnmt1-2* and *dxo1* genotypes. **b** PCA of the transcriptomes from WT, *rnmt1-2, dxo1, 35Spro:RNMT1* and *dxo1/35Spro:RNMT1*. The RNA-seq data were from four biological replicates. Top ten GO terms of up- and downregulated DEGs caused by *rnmt1-2* (**c**) and *dxo1* (**d**). The dotted line indicates the FDR-adjusted *p* value = 0.05 (**c**, **d**).

To reveal how the defect in m^7^G capping caused by the *rnmt1-2* and *dxo1* mutations might affect transcriptome profiles, RNA-sequencing (RNA-seq) was carried out to compare transcriptomes of 12-day-old seedlings of WT, *rnmt1-2*, *dxo1*, *35Spro::RNMT1*, and *dxo1/35Spro::RNMT1* lines. Genes whose transcript levels differed significantly (FDR-adjusted *p* value *<* 0.05) and by at least two-fold were further analyzed as differentially expressed genes (DEGs) (Supplementary Data 1). In comparison with WT, 1006 DEGs were upregulated and 646 downregulated in *rnmt1-2* (Fig. 6a). In *dxo1*, 1909 DEGs were upregulated and 1180 downregulated, indicating a more profound effect on gene expression by the *dxo1* mutation than by *rnmt1-2*, which is consistent with the more severe morphological defects in *dxo1* compared to *rnmt1-2* (Fig. 4e). Among the upregulated genes in *rnmt1-2*, 75% were also upregulated in *dxo1*. Among the downregulated genes in *rnmt1-2*, 60% were also down-regulated in *dxo1* (Fig. 6a and Supplementary Data 1). The results indicated that the sets of genes affected by *rnmt1* are mostly affected by *dxo1*, particularly among the upregulated genes. Principal component analysis (PCA) also shows that the *dxo1* transcriptome was similar to the *rnmt1* cluster, and the change of the transcriptome profile caused by *dxo1*, in comparison with WT, is more extensive than that caused by *rnmt1* (Fig. 6b). Overexpression of *RNMT1* in *dxo1* (*dxo1/35Spro::RNMT1*) largely complemented the transcriptome variation caused by the *dxo1* mutation, whereas overexpression of *RNMT1* in WT had a minor effect on overall gene expression profile (Fig. 6b and Supplementary Fig. 10).

Gene Ontology (GO) enrichment analysis (Supplementary Data 2) of the upregulated DEGs showed that genes in the functional categories of protein synthesis (ribosomal biogenesis and translation) were most significantly enriched by both *rnmt1-2* and *dxo1* mutations; however, the *dxo1* mutation also upregulated sets of genes involved in biotic and abiotic stress responses (such as responses to hypoxia and salicylate) (Fig. 6c, d). Among the down-regulated genes, the *dxo1* mutation most profoundly affected genes in photosynthesis-related functions, whereas the *rnmt1* mutation decreased expression of genes in the categories of transcription and stress responses (Fig. 6c, d). The direct comparison between the *dxo1* and *rnmt1-2* transcriptome profiles also show that photosynthesis-related genes were downregulated, whereas stress response genes were up-regulated in *dxo1* (Supplementary Fig. 11). Together, the transcriptome data indicate that expression of shared or similar sets of genes were affected by both *rnmt1-2* and *dxo1* mutations, particularly upregulation of genes for protein synthesis; however, DXO1 might have an additional role, loss of which causes downregulation of photosynthesis genes but up-regulation of stress response genes.

## Discussion

Growing evidence has shown that the RNA capping process is modulated to actively regulate gene expression in animals, and that conversion of the G cap to the m^7^G cap could be differentially regulated in a gene-specific manner in response to signals^11,13^. Recent findings on various non-canonical caps, such as the NAD cap, further indicate the complexity of RNA capping and decapping in gene regulation. In this work, we have identified RNMT1 as the enzyme that catalyses conversion of the G cap to the m^7^G cap in Arabidopsis and found that DXO1 functions as an activator of RNMT1. The mammalian RNMT is activated by the small protein RAM^6^. The *Vaccinia* poxvirus encodes a single protein (D1) that catalyses both addition of the G cap and its methylation; however, its cap methyltransferase activity is activated by binding to another subunit (D12)^36,37^. DXO1 is evolutionally unrelated to RAM or D12, suggesting that distinct mechanisms have evolved for activating the cap methyltransferases. As a small protein, RAM has not been reported to have another function. The DXO family proteins in yeast and mammals are enzymes that hydrolyze the G cap and the m^7^G cap, and recently some of them were found to also decap the NAD cap (deNADding)^14,16,25^. However, Arabidopsis DXO1 possesses the deNADdding activity but does not hydrolyze the G or m^7^G cap^26,27^. The previous reports from us and others indicate that, in addition to its deNADing activity, DXO1 has another important non-enzymatic function that is responsible for most of the phenotypes associated with the *dxo1* mutation^26,27^. This study reveals that the plant DXO protein is a critical component in m^7^G capping.

Our conclusion on the role of *DXO1* in the m^7^G capping process is supported by multiple lines of evidence: association of DXO1 with RNMT1, which increases the RNA cap methyltransferase activity of RNMT1; the phenotypic and transcriptomic similarities of the *dxo1* and *rnmt1-2* mutants; partial restoration of the *dxo1* phenotypes by overexpression of RNMT1; and the defect in G cap methylation in plant cells caused by both mutations. Therefore, in addition to its deNADding activity, the plant DXO1 has evolved with the unique function as an activator of the cap methyltransferase. This unique function of DXO1 was apparently evolved through its plant-specific N-terminal extension, which alone can bind to and activate RNMT1. As expected, RNMT1 and DXO1 interact in the nucleus; however, RNMT1 and DXO1 are also present and interact in the cytosol. The m^7^G capping machinery in mammalian cells are also known to be present in the cytosol for mRNA recapping^9^, raising the possibility that mRNA in plants could also be recapped in the cytosol.

DXO1 is predicted to have a putative RNA-binding region in its catalytic domain (Supplementary Fig. 1), and our previous study also suggests that it binds to RNA bodies^26^. Its interaction with RNMT1 while binding to RNA could enhance the recruitment of RNA to RNMT1 for more efficient cap methylation in a manner similar to activation of the mammalian RNMT by RAM^6^. As DXO1 also enhances RNMT1’s cap methyltransferase activity when GpppA and GpppG were used as the substrates, it is also possible that its interaction with RNMT1 might directly enhance the cap methyltransferase activity by altering the RNMT1’s structure. In addition to function in regular m^7^G capping, our findings also raise a possibility that NAD-RNA decapping might be connected with m^7^G capping in plants.

The loss-of-function mutations of the mammalian DXO and the Arabidopsis DXO1 have been reported to increase the levels of NAD-capped RNAs^16,24^. However, we have recently found that at least some NAD-RNAs in eukaryotic cells previously identified and reported are likely noise from m^7^G-RNAs^20,23^, and therefore the effect of the mutations of the DXO family proteins on NAD-RNA profiles in the mammals and Arabidopsis remains to be further defined.

As the m^7^G cap stabilizes mRNAs^38^, G-capped mRNAs are expected to be less stable. However, the defect in cap methylation caused by the *rnmt1* and *dxo1* mutations had different effects on transcript levels for different genes. It is possible that the *rnmt1-2* and *dxo1* mutations might affect RNA cap methylation more for some genes than for others. Similarly, it was reported that a defect in cap methylation in humans did not affect the transcript maintenance of all genes^6^. Our results show that both the *rnmt1-2* and *dxo1* mutations cause increased transcript levels of genes involved in protein synthesis. In addition to a direct effect of defective cap methylation by these mutations on transcript maintenance, the increased expression of genes involved in protein synthesis could also be due to an indirect effect. As the *rnmt1-2* and *dxo1* mutations lead to a reduction of m^7^G-capped mRNAs but an increase in G-capped mRNAs and as G-capped mRNAs are likely less effective in translation^6^, the mutant cells might upregulate the genes in protein synthesis as an attempt to compensate for reduced protein synthesis. However, the effect of the *dxo1* mutation on downregulation of photosynthesis-related genes and upregulation of stress-responsive genes was not obviously displayed in the *rnmt1* mutant and could not be complemented by overexpression of RNMT1 in *dxo1*. It is possible that this additional effect of the *dxo1* mutation could be due to the loss of other function(s) of DXO1, such as its deNADding function. Further study to reveal mechanistic details of the interactions between DXO1 and RNMT1 and the possible interconnection of NAD decapping with m^7^G capping would be greatly helpful in fully understanding gene regulation by RNA capping and decapping.

## Methods

### Plant materials and growth conditions

Arabidopsis *thaliana* Columbia (Col-0) ecotype was used as the wildtype in this study. The T-DNA insertion line (SAIL_1252_D05; *rnmt1-1*) in the Col-0 background was obtained from the Nottingham Arabidopsis Stock Center (NASC) (http://arabidopsis.info/). The *dxo1* (*dxo1-1*) mutant used in this study was previously reported^26^.

To grow Arabidopsis on agar plates, seeds were surface-sterilized and placed on the half-strength Murashige and Skoog (MS) Basal Salts medium (Sigma-Aldrich) with 0.05% MES, 2% (w/v) sucrose, and 0.8% agar with pH adjusted to 5.7. The Petri dishes were incubated at 4 °C in darkness for 3 days before being placed in a growth room. Nine days later, the seedlings were transferred to soil. Seedlings were grown at 22 °C under a 16 h light/8-h dark photoperiod and a light intensity of 125 mol m^−2^ s^−^^1^ provided by cool white fluorescent lamps. In some experiments, seeds were germinated on soil, and two-week-old seedlings were transplanted for further growth.

### Oligonucleotide primers

The sequences and other information on the primers used in the study are listed in Supplementary Table 2.

### Arabidopsis transformation

The binary plasmids (1–2 μg) were transformed into *Agrobacterium* strain *GV3101*. Selected transformed colonies were confirmed by colony PCR. The *Agrobacterium* strains were used to transform Arabidopsis by using the floral dipping transformation method^39^.

### Genotyping T-DNA insertion lines

PCR of genomic DNA was carried out to detect the *rnmt1-1* and *dxo1-1* alleles using the T-DNA left border primer LB1 or LBb1 and the gene-specific primer RP. For amplification of the wild-type allele, the primer pair LP and RP was used. PCR was performed using Taq DNA Polymerase (Takara) according to the manufacturer’s instruction.

### Yeast two-hybrid library screening and verification

For the yeast two-hybrid screening, the full-length open reading frame of *DXO1* was cloned into pGBKT7 (Takara) to generate BD-DXO1 fusion protein as the bait. The Mate & Plate™ Universal Arabidopsis Library (Takara) was screened according to the Matchmaker™ Gold Yeast Two-Hybrid System User Manual (Takara). The bait strain expressing *BD-DXO1* was mated with the Y187 library strains. Zygotes were spread on the selective medium without leucine (-Leu), tryptophan (-Trp), histidine (-His) and adenine (-Ade), and colonies were patched out onto the higher stringency medium supplemented with the toxic drug Aureobasidin A (AbA) and X-α-Gal. Blue colonies that grew on the selective medium were picked up, and Matchmaker Insert Check PCR Mix 2 (Takara) was used to amplify the Arabidopsis cDNA inserts fused with AD. The PCR products were sequenced to identify the inserts.

To analyze interactions between known proteins using the Y2H system, one protein was fused with the BD domain in pGBKT7 and the other into pGADT7 to generate AD-domain fusion protein. The BD and AD fusion constructs were introduced into the yeast strain Y2H Gold (Takara) to detect their interactions. Selective medium (-Leu), (-Trp), (-His), (-Ade), supplemented with AbA (and X-α-Gal in some analyses) was used to determine protein interactions.

### Bimolecular fluorescence complementation (BiFC) assay

The *DXO1* open reading frame was inserted into the pSY736 (nYFP) vector^40^ to generate *DXO1-nYFP*, and the RNMT1 coding sequence was cloned into pSY735 (cYFP) to generate *RNMT1-cYFP*. Plasmids (5 μg each) were co-transfected into the WT protoplast^41^, and YFP signal was detected 8 h after transfection by confocal microscopy with 514 nm excitation wavelength laser.

### Co-immunoprecipitation (Co-IP) assay

Protoplasts from leaves of WT or the *35S:DXO1-FLAG* transgenic plants were transfected with the *35S:DXO1-FLAG* plasmid made by inserting *DXO1* open reading frame into *pCambia1305-35S* plasmid. Plasmids (100 μg) carrying the *RNMT1* or *FCA* fusion construct *pHBT-35Spro::RNMT1cds-HA* or *pHBT-35Spro::FCAcds-HA* were transfected into 1 mL (~1 × 10^6^ cells) protoplasts from 3-week-old plants according to the previously reported procedure^40^. Ten hours after transfection, protoplasts were harvested and lysed in 200 μL of the immunoprecipitation (IP) buffer [150 mM NaCl, 50 mM Tris-HCl, pH 7.5, 10% glycerol, 0.5% Triton X-100, and Protease Inhibitor Cocktail (Sigma) for plant cell extracts] with or without 50 μg RNase A (Thermo). For each sample, 50 μL of lysate was kept as the input. An extra 300 μL of immunoprecipitation buffer was added into the remaining lysate and vigorously vortexed. The supernatant was incubated with 20 μL of anti-FLAG M2 Affinity Gel (Sigma) for 2 h at 4 °C. The resin was washed four times with the immunoprecipitation buffer supplemented with 1% Triton X-100. The resins were boiled in 50 μL of SDS-PAGE loading buffer to obtain the elute. The proteins were detected by Western blotting using the anti-FLAG M2 antibody (Sigma) or the anti-HA antibody (Sigma). For co-IP in plants, 12-day-old seedlings of WT and *DXO1pro::DXO1-FLAG* transgenic lines^26^ were harvested. Proteins were immunoprecipitated as described above. RNMT1 in the precipitate was detected by Western blotting using the anti-RNMT1 polyclonal rabbit antibodies that were raised by immunization with a recombinant protein fragment corresponding to the amino acids 220-370 of RNMT1 (ABclonal, Shanghai, China).

### Plasmids construction

To overexpress RNMT1, its cDNA fragment was cloned into the *pCambia 1305-35S* binary vector to generate the *35Spro:RNMT1* construct, which was then introduced into *Agrobacterium* strain *GV3101* and used for transformation of the *dxo1* mutant and WT plants.

For using genome editing to generate *rnmt1* mutants, we used the CRISPR/cas-9 plasmid pHEE2E-TRI^42^. Col-0 plants were transformed with this plasmid carrying two sgRNA sequences, aacgggtggattttatagAG and GGAGCGGGCGGAGGAAGAAT, derived from the last intron and exon of *RNMT1*. T1 plants were analyzed by PCR using the *rnmt1*-mutant-F and *rnmt1*-mutant-R primer pair, and the PCR products were sequenced to identify mutations. The T1 plants containing a mutated allele were self-fertilized, and homozygous T2 mutants were identified by PCR and sequencing of the PCR products. The progenies without the Cas9 construct were used for further analysis.

### Proteins expression in *E. coli* and purification

The *RNMT1*, *DXO1, nDXO1*, and *cDXO1* coding sequences were PCR-amplified from cDNA of WT, and the DXO1^K412Q^ mutant clone was previously reported^26^. These fragments were cloned into the pET28a expression vector to generate the expression constructs. The *GST-DXO1* fusion was cloned into the pGEX-4T-1 vector. The plasmids were transformed into *E. coli* BL21 Rosetta cells to express recombinant protein fused with the 6X His tag or GST tag at their N-terminus. To induce expression of these proteins, IPTG was added to cells when OD_600_ = 0.6, and the cells were incubated at 28 °C for 3–4 h. The recombinant proteins were purified by Ni-NTA or GST agarose beads according to the manufacturer’s instruction (Thermo Fisher). The purified recombinant proteins were stored in a buffer containing 20 mM Tris (pH 7.5), 100 mM NaCl and 5% (v/v) glycerol at −20 °C until further use.

### In vitro GST pull-down

Two μg GST-RNMT1 and His-DXO1 or His-cDXO1 were incubated with 40 μL GST agarose beads in the 500 μL GST reaction buffer containing 20 mM Tris-HCl, pH7.5, 100 mM NaCl, 1 mM DTT and 1 mM EDTA for 1 h. After washing 4 times with the GST buffer, proteins were eluted by 10 mM GSH. Eluted proteins were detected by Western blotting using the anti-His antibodies (Sigma) or the anti-GST antibodies (Cell Signaling Technology).

### LC-MS/MS analysis

Ultra-high performance liquid chromatography coupled with a Triple-Stage Quadrupole Mass Spectrometer (Thermo) was used to identify and quantify GpppA, m^7^GpppA, GpppG, m^7^GpppG, GDP, and m^7^GDP. Purchased standard chemicals were used for generating the standard curves for normalization of the relative peak areas (Supplementary Fig. 12). An injection volume of 10 μL of the sample was separated by a 2.1 mm × 100 mm ACQUITY UPLC BEH C18 column (1.7 μm particle size, Waters Corporation) within a 15 min gradient. 0.1% NH4OAc solution (A) and pure methanol (B) were used as mobile phases to elute the analyte at a flow rate of 0.3 mL/min: 0–4 min, 2% B; 4–11 min, 2–100% B; 11–12 min, 100% B; 12–12.2 min, 100% to 2% B; 12.2–15 min, 2% B. A scan range from 70 to 1000 m/z was used to do MS1 acquisition in positive mode with the resolution of 70000 and the AGC target of 1e6. A selective reaction monitoring (SRM) assay was performed in ESI + mode to target-detect the precursor ion of GDP (m/z = 444.0316 → 152.057), m^7^GDP (m/z = 458.0478 → 166.072), GpppA (m/z = 773.0841 → 136.061), m^7^GpppA (m/z = 787.0790 → 136.061), GpppG (m/z = 789.0790 → 248.077) and m^7^GpppG (m/z = 803.0947 → 248.077). GDP and m^7^GDP standards were purchased from Santa Cruz Biotechnology, while GpppA, GpppG, m^7^GpppA and m^7^GpppG were from NEW ENGLAND BIOLABS. For MS2 acquisition, the other parameters are as follows: resolution of 17500, an isolation window of 0.4 m/z, and a collision energy of 10, 20, and 39 ce.

### Cap methyltransferase activity assay

The 29-nt GpppA-RNA and GpppG-RNA used for the RNMT1 activity assay was prepared as reported previously^21,43^ with minor modification. Briefly, a double-stranded DNA template was formed by annealing two single-stranded DNAs (5′ CAG**TAATACGACTCACTATT**ATTTTCGTTGTTGTTCTGTTTTGCCTTGG3′ and 5′ CCAAGGCAAAACAGAACAACAACGAAAATAATAGTGAGTCGTAT TACTG 3′ for GpppA-RNA synthesis, and 5′ CAG**TAATACGACTCACTATA**GAATACAATCAACATCTCTTCACCCTCCC 3′ and 5′ GGGAGGGTGAAGAGATGTTGATTGTATTCTATAGTGAGTCGTATTACTG 3′ for GpppG-RNA synthesis, with the promoter sequence in bold and the transcription start site underlined. The templates were used in in vitro transcription by T7 polymerase with GpppA and pppG as the initiation nucleotide to synthesize 29-nt GpppA-RNA and pppG-RNA, respectively. The products were extracted by the RNA Clean & Concentrator-5 kit (Zymo Research), purified by Micro Bio-Spin™ P-30 Gel Columns (Bio-rad) to remove small molecules, and eluted with RNase-free water. To generate GpppG-RNA, the G cap was added to pppG-RNA by Vaccinia Capping Enzyme (NEW ENGLAND BIOLABS) in presence of GTP but no SAM in the reaction, and the products were purified by extraction using the RNA Clean & Concentrator-5 kit (Zymo Research).

For cap methyltransferase assays using the RNA analogs GpppA or GpppG (NEW ENGLAND BIOLABS) as the substrate, the reaction was conducted at 37 °C for 3 h in a 250 μL reaction mixture containing 50 μM GpppA or GpppG, 250 nM RNMT1 (with or without 250 nM DXO1, 250 nM nDXO1, 250 nM cDXO1, or 250 nM DXO1^K412Q^), 250 nM SAM, 10 mM Tris-HCl (pH 7.5), 100 mM KCl, 2 mM MgCl_2_, 2 mM Dithiothreitol (DTT), and 1 U (1 μl) Murine RNase inhibitor. The cap methylation reaction with the 29-nt RNA was carried out in the solution with 50–100 nM GpppA-RNA or GpppG-RNA, 6.4 μM SAM, 50 nM RNMT1 with or without DXO1 for 30 min. After the reaction, GpppA or GpppG and their methylation products were purified by extracting with acid phenol and chloroform and eluted with a 5 μL final volume by ethanol and linear acrylamide. The 29-nt GpppA-RNA and its products were purified by the RNA Clean & Concentrator-5 kit (Zymo Research) and eluted with a 15 μL final volume.

For detection of the m^7^G cap by dot blotting, an aliquot of 2.5 μL of the GpppG or GpppA reaction product was spotted on a nylon membrane. After UV cross-linking, the anti-m^7^G antibody (MBL) was used to detect the m^7^G cap. Another aliquot of 2 μL product was diluted by RNase-free water to 70 μL and subjected to mass spectrometry analysis to detect and quantify GpppA/G and m^7^GpppG/A. For measuring the products from the 29-nt G-capped RNA, GpppA and m7GpppA were released from RNA by digesting 10 μl of the reaction product with nuclease P1 at 37 °C for 2 h before the mass spectrometry analysis. For detecting the 29-nt m^7^GpppA-RNA by RNA blotting analysis, an aliquot of 5 μL of reaction products from GpppA-RNA and GpppG-RNA were separated by polyacrylamide gel electrophoresis, blotted onto a nylon membrane, and detected by the anti-m^7^G antibody (MBL).

### Observation of RNMT1-GFP subcellular localization

To generate the *35S:RNMT1-GFP* construct, the *RNMT1* gene was PCR-amplified from Col-0 genomic DNA and inserted into the vector pA7-GFP. The construct was transformed into the *rnmt1/+* plants, and some progenies carrying the construct and homozygous for *rnmt1* were identified. Preparation of Arabidopsis protoplasts and PEG-mediated plasmid DNA transfection was carried out as previously described^41^. The transfected protoplasts were kept at room temperature for overnight under light to express RNMT1-GFP. The fluorescent signal was detected under confocal microscopy (Leica SP5II Confocal System). A laser with a 488 nm excitation wavelength with a 505/530 nm filter was used to observe the GFP signal.

### Observation of pollen, ovules, and developing embryos

Anthers were collected from *rnmt1*/+ and WT plants and placed in droplets of modified Alexander staining solution^44^. After staining for at least 10 min, pollen viability was observed under a dissecting microscope.

For whole mount observation of developing seeds, seeds at different developmental stages were excised from siliques and fixed in ethanol/acetic acid (6:1) for 2 h at room temperature. After several washes with a gradient of ethanol (from 100% ethanol to 70% ethanol), embryos were mounted in a chloralhydrate/glycerol/water (8:1:2) mixture and cleared for one hour at room temperature. Differential interference contrast (DIC) images were obtained using a Leica confocal microscope (Leica SP5II Confocal System). To observe ovules and developing embryos using scanning electron microscopy (SEM), the samples were prepared according to the previously described method^45^. The prepared samples were mounted on stubs for gold-palladium coating and examined with a Scanning electron microscope (SEM).

### Quantification of the G cap and m^7^G cap in cellular RNA samples

Total RNA of WT, *dxo1*, *rnmt1-2*, *rnmt1-3*, and *dxo1/DXO1*^K412Q^^26^ was isolated from 4-week-old plants using PureLink Plant RNA Reagent (Ambion) according to the manufacturer’s instruction. The mRNA was enriched from 1 mg of sample using Oligo d(T)25 magnetic beads (NEW ENGLAND BIOLABS). The amount of mRNA was measured by Invitrogen qubit.

Four μg mRNAs were digested by ‘mRNA Decapping Enzyme’ (NEW ENGLAND BIOLABS) to release m^7^GDP and GDP, which were quantified by mass spectrometry. The mRNA sample without decapping enzyme treatment were used as a negative control. For RNA blotting analysis, the anti-m^7^G antibodies were incubated with mRNA blots spotted with 200 ng mRNAs for 1 h at room temperature.

### Reverse transcription-quantitative PCR and RNA sequencing

Total RNAs were isolated from 12-day-old Arabidopsis plants using the Agilent Plant RNA Isolation Mini Kit (Agilent Technologies) for qPCR or RNA-seq. For qPCR, Genomic DNA was removed by treating 1 μg purified RNA with gDNAse Eraser at 42 °C for 15 min. cDNA was synthesized using PrimeScript RT Reagent Kit with gDNA Eraser (Takara) according to the manufacturer’s instruction. The cDNA samples, the specific primers, and 2× UltraSYBR Mixture (CWBIO) were added to a 25 μL reaction mixture for PCR. Three or four biological replicates were included. The *UBQ5* or *ACTIN2* gene was used as an internal control. The PCR reaction was conducted using the Applied Biosystems StepOne Plus real-time PCR machine (Thermo). For RNA-seq, RNA samples were prepared as described above and cDNA libraries were prepared by Novogene (Beijing) using the NEBNext Ultra RNA library prep kit for Illumina sequencing (NEW ENGLAND BIOLABS) according to the manufacturer’s instruction.

### Bioinformatics analysis of RNA-sequencing data

Next generation sequencing data was generated by Illumina NovaSeq using 150-bp paired-end sequencing. The raw data were filtered by SOAPnuke^46^ to remove reads with adaptors or low-quality sequences. The clean data were aligned to the Arabidopsis *thaliana* genome (TAIR10) using HISAT2^47^. Expression levels of genes were calculated by HT-seq^48^. Differential gene expression analysis was conducted using DESeq2, and DEGs with at least two-fold change and FDR-adjusted *p* value < 0.05 were further analyzed^49^. GO term enrichment analysis was performed using DAVID^50^.

### Statistical analysis

When conducting multiple comparisons, One-way ANOVA analysis with Tukey’s test was used to indicate significant differences *P* < 0.05 with different letters. Two-tailed unpaired *t*-tests were performed to compare two groups. ** represents *P* < 0.01. Chi-square test was used to analyze segregation ratios of different genotypes.

### Reporting summary

Further information on research design is available in the Nature Portfolio Reporting Summary linked to this article.

## Supplementary information

Supplementary information

Description of Additional Supplementary Files

Supplementary Data 1

Supplementary Data 2

Reporting Summary

## Data Availability

The RNA-seq data have been submitted to Sequence Read Archive (SRA) of the National Center for Biotechnology Information and BioProject ID is PRJNA799165. Source data are provided with this paper.

## Source data

Source data
