# Supplementary material for: Arabidopsis DXO1 activates RNMT1 to methylate the mRNA guanosine cap
Source: Nat Commun. 2023 Jan 13;14:202. doi: 10.1038/s41467-023-35903-8 (PMC9839713; doi:10.1038/s41467-023-35903-8)
Supplement: Supplementary file 1 — Supplementary information [file 41467_2023_35903_MOESM1_ESM.pdf]

Arabidopsis DXO1 activates RNMT1 to methylate the mRNA guanosine cap

a

Predicted disordered regions (shown in green) in DXO1

MDSPPKKNPm ddlfg**EDSDN DSRSSRSKSS SSGNASSSSS SSSSSSSSSS AAGGDGEGDG GGADSGSASD SGSGSSGGKE EHGDDKVESY**
RSNDNGESGV YPYEEEEEEE EDEKDLFGsd neeytktpai stysipvlpa gwsndnhggr ggmgrgrwsn grggpgllpr pgpypgrggn
rggfggryqn yqrderfvse lklsksketl arkqtnfqep celtcysrve ggeviyddqg lrlfkrhige eigadlnqgy dtfiekkdlg
segfgdllgs iraknisldn ihfvtfrnnl nkilgaaynr hepwemgvhk rngtiyldvh klperpqsdl drrrcywgyc feslatedpg
raygeeihhv danvefcsvv rtklgahrvm mgaemdccdv sdkgkrfyve lkttrelddr tvdrferekl lkfwiqsfva gvpyivvgfr
ddggrlvrte rlttrdiahr arlknywqgg vclafadevl cwlygtvken edyilqfvhp fmrlellqaq scpdaitnhv hllq**NPASPP**

b

Predicted protein-binding region (s) in DXO1(1 represents the putative disordered protein-binding residues; 0 otherwise)
111111111111111111111111111111111111111111111111111111111111111111111111111111111111111111111111111111111111111111111111111111111111
111000000000000000000000000000000000000000000000000000000000000000000000000000000000000000000000000000000000000000000000000000000000
000000000000000000000000000000000000000000000000000000000000000000000000000000000000000000000000000000000000000000000000000000000000
000000000000000000000000000000000000000000000000000000000000000000000000000000000000000000000000000000000000000000000000000000000000
00000000000000000000000000000000000000000000000000000000000000000000000000000000000001

c

Predicted RNA-binding region(s) in DXO1 (1 represents the putative disordered protein-binding residues; 0 otherwise)
000000000000000000000000000000000000000000000000000000000000000000000000000000000000000000000000000000000000000000000000000000000000
000000000000000000000000000000000000000000000000000000000000000000000000000000000000000000000000000000000000000000000000000000000000
000000000000000000000000000000000000000000000000000000000000000000000000000000000000000000000000000000000000000000000000000000000000
000000000000000000000000000000000000000000000000000000000000000000000000001101111111111111111111100000000000000111111111111111111111
11111111111110000000001111111100000000000000000000000000000000000000000000000000000000

Supplementary Fig. 1. Predicted intrinsically disordered regions and protein- or RNA-binding regions in DXO1. The intrinsically disordered regions (**a**) of DXO1 were predicted by the disEMBL algorithm (http://dis.embl.de/) and the protein- (**b**) and RNA-binding (**c**) regions were predicted using the DisoRDPbind algorithm (http://biomine.cs.vcu.edu/servers/DisoRDPbind/#Help)

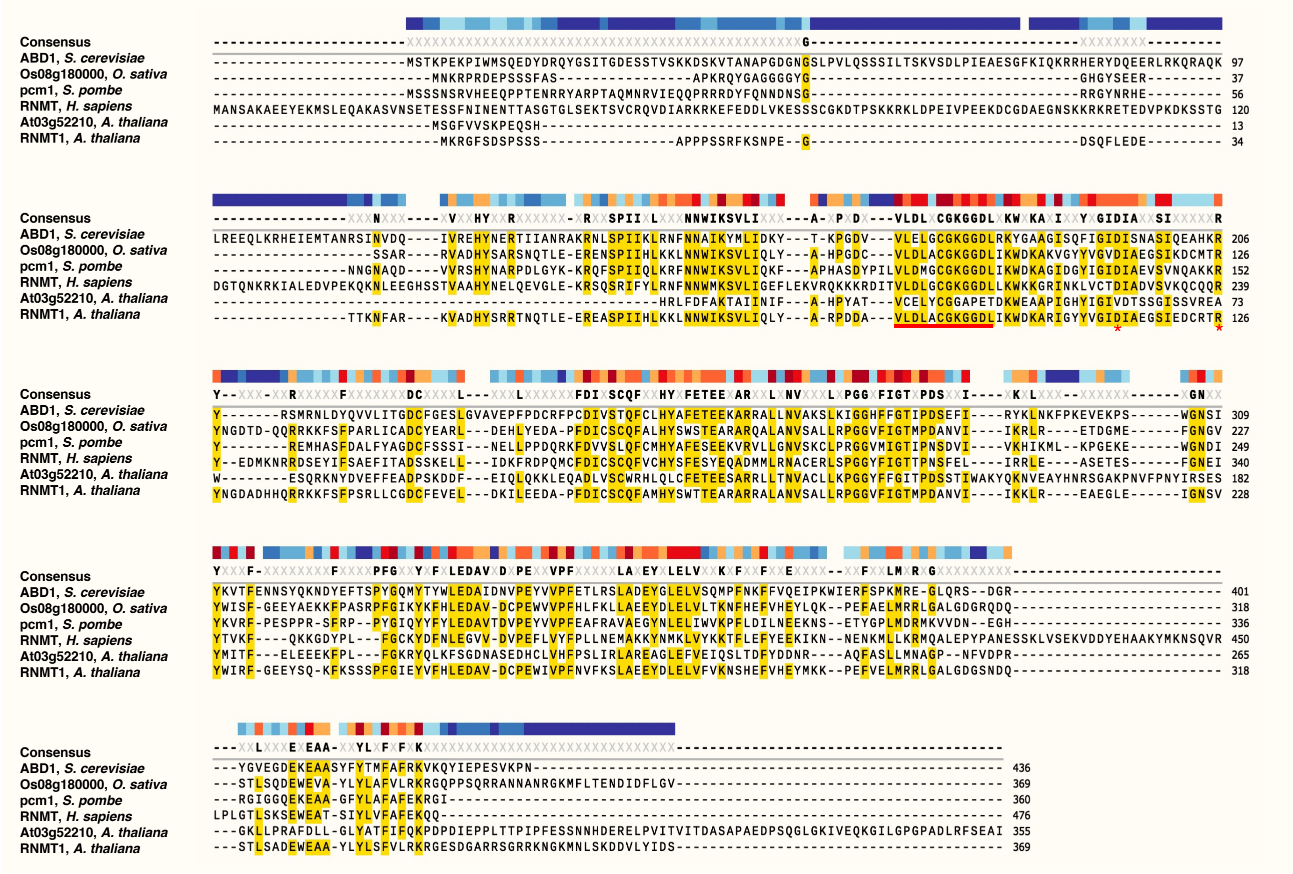

Supplementary Fig. 2. Sequence conservation between RNMT1 and the RNA cap methyltransferases from yeast and humans. A multiple sequence alignment between RNMT1, At3g52210, the rice homolog (Os08g0103), and known RNMTs from budding yeast (ABD1), fission yeast (Pcm1), and humans (RNMT). The residues that are highly conserved are shown in yellow shadow. The highly conserved VLxI/LxxGxGxDL motif and two of the highly conversed residues known to be critical for the cap methyltransferase activity are indicated by the red line and red stars, respectively.

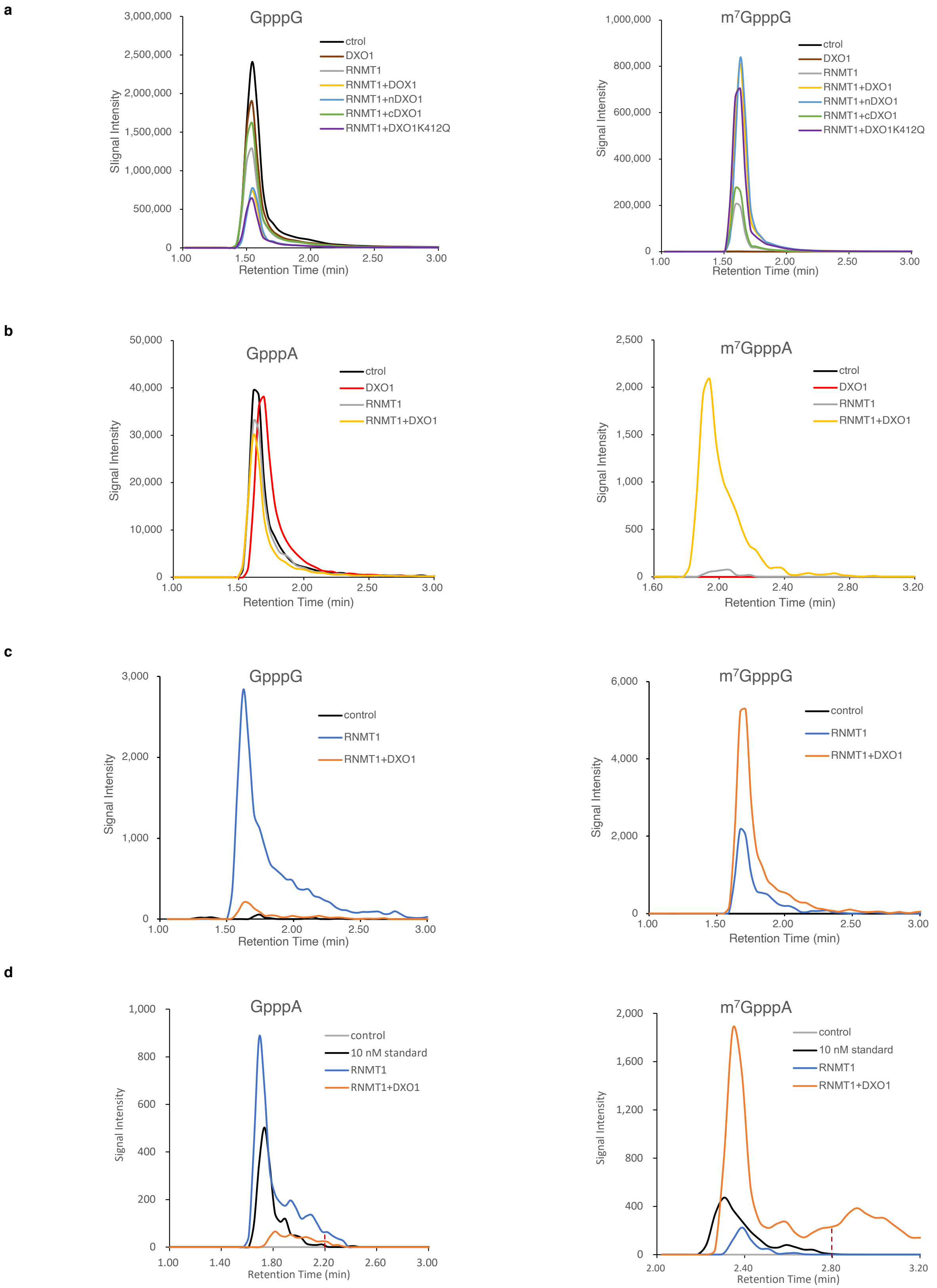

Supplementary Fig. 3. LC-MS peak plots of GpppG/A and m^7GpppG/A corresponding to their qualification data shown in Fig. 3. Conversion of GpppG (**a**), GpppA (**b**), GpppG-RNA (29-nt, **c**) and GpppA-RNA (29-nt,**d**) to m^7GpppG (**a**), m^7GpppA (**b**), m^7GpppA-RNG (29-nt, **c**) and m^7GpppA-RNA (29-nt, **d**), respectively by RNMT1 alone or with DXO1, nDXO1, cDXO1 or DXO1K412Q. Capped-RNAs were digested with nuclease P1 to release GpppG/A and m^7GpppG/A. Control: the reaction with no protein. The X axis indicates retention time and Y axis indicates signal intensity. For quantification of GpppA and m7GpppA in **d**, we used the same range of retention times as that of the standard (1.60-2.20 min for GpppA and 2.20-2.80 min for m^7GpppA). Three biological replicates were included in the analysis with similar results, and one of the three replicates was shown in this figure.

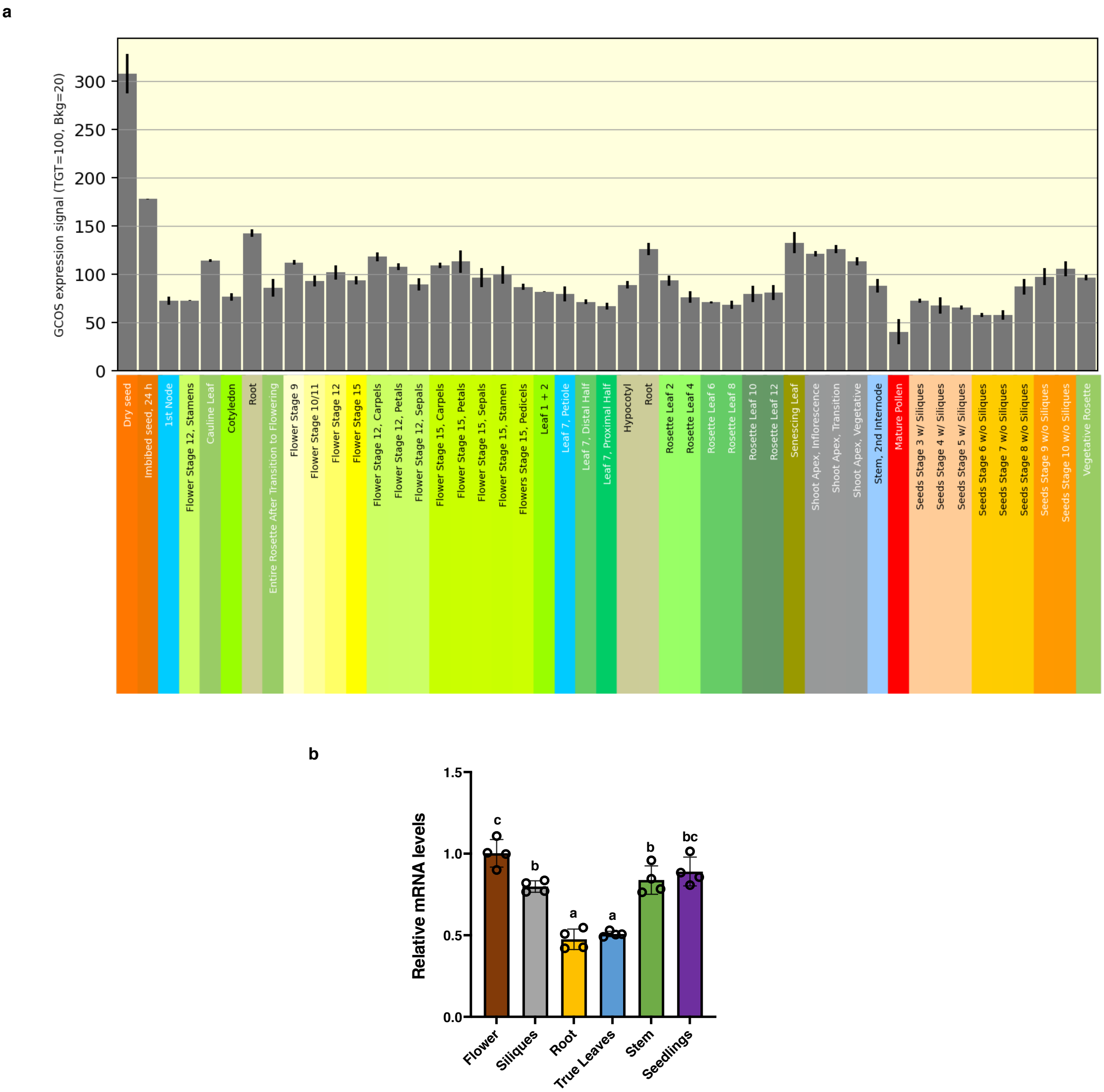

Supplementary Fig. 4. *RNMT1* is a housekeeping gene. a, *RNMT1* relative transcript levels in different organs based on the transcriptome data in the *Arabidopsis* eFP Browser database. **b**, *RNMT1* transcript levels in different organs determined by quantitative real-time PCR. Different letters indicate significant differences by one-way ANOVA, Tukey's test (*p<0.05*). Data are mean± SD (n=4 biologically independent plants). *ACTIN2* was used as the internal control.

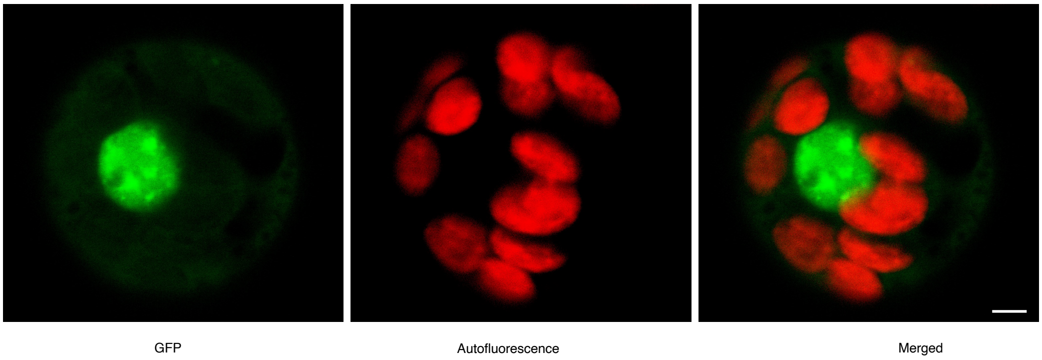

Supplementary Fig. 5. RNMT1-GFP is localized in both the nucleus and the cytosol. RNMT1-GFP was localized in the nucleus of Arabidopsis protoplasts transiently expressing *35S*:*RNMT1-eGFP* fusion gene. The experiments were independently performed three times with similar results. Bar: 2.5 µM.

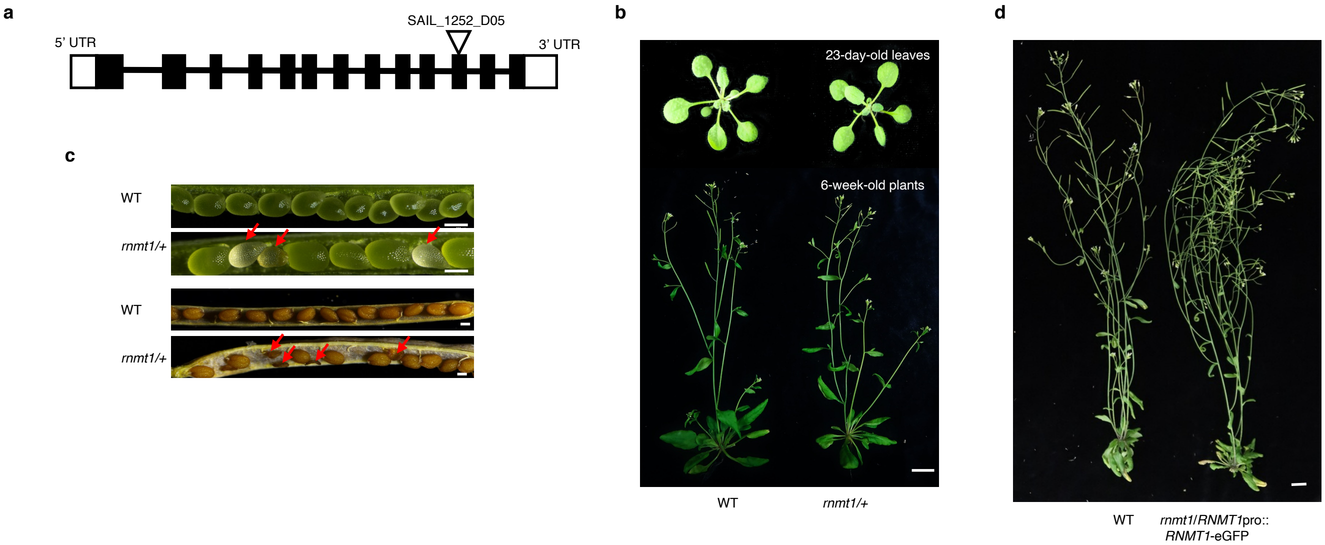

Supplementary Fig. 6. The *rnmt1* mutation leads to seed abortion. a, Schematic representation of the *RNMT1* gene structure. The triangle points to the T-DNA insertion site. Exons and introns are represented by black boxes and lines, respectively. **b**, Phenotypes of WT and heterozygous *rnmt1/+* plants. **c**, Opened siliques of self-pollinated WT and *rnmt1/+* plants at fully expanded and mature stages. Abnormal seeds are indicated by red arrows. **d**, Complementation of the *rnmt1* mutant by *RNMT1*pro:*RNMT1*-eGFP. 7-week-old plants of WT and the homozygous *rnmt1* plant carrying the *RNMT1-GFP* transgene. Bar: 1 cm (**b**,**d**), 250 µm (**c**).

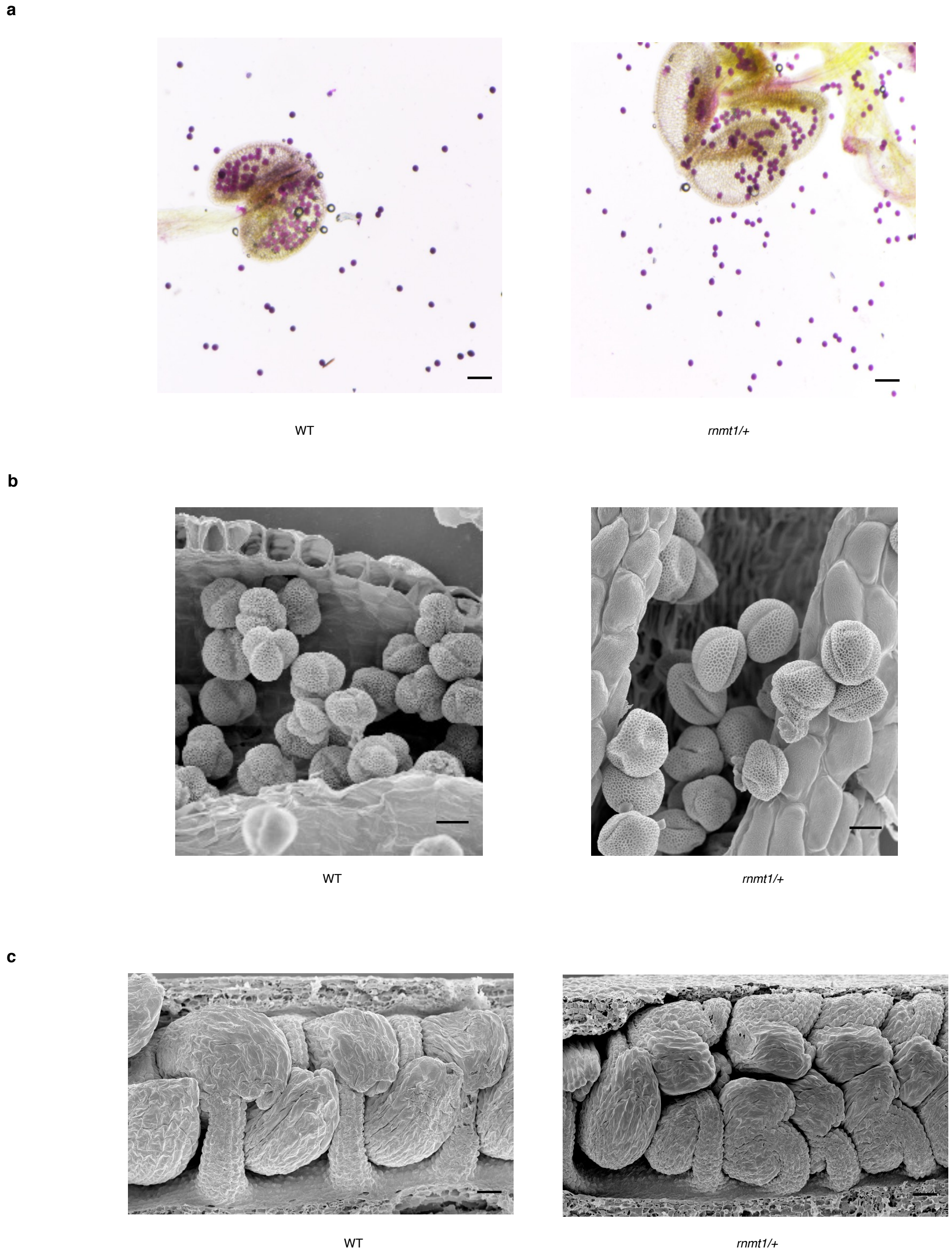

Supplementary Fig. 7. Pollen and ovule development of the *rnmt1/+* plants is normal. a, Viability staining of pollen from WT and *rnmt1/+* plants. **b**, Scanning electron micrographs of pollen from WT and *rnmt1/+* plants. **c**, Scanning electron micrographs of ovules from WT and *rnmt1/+* plants. The experiments in **a-c** were independently performed three times with similar results. Bars: 100 µm (**a**), 10 µm (**b, c**).

a

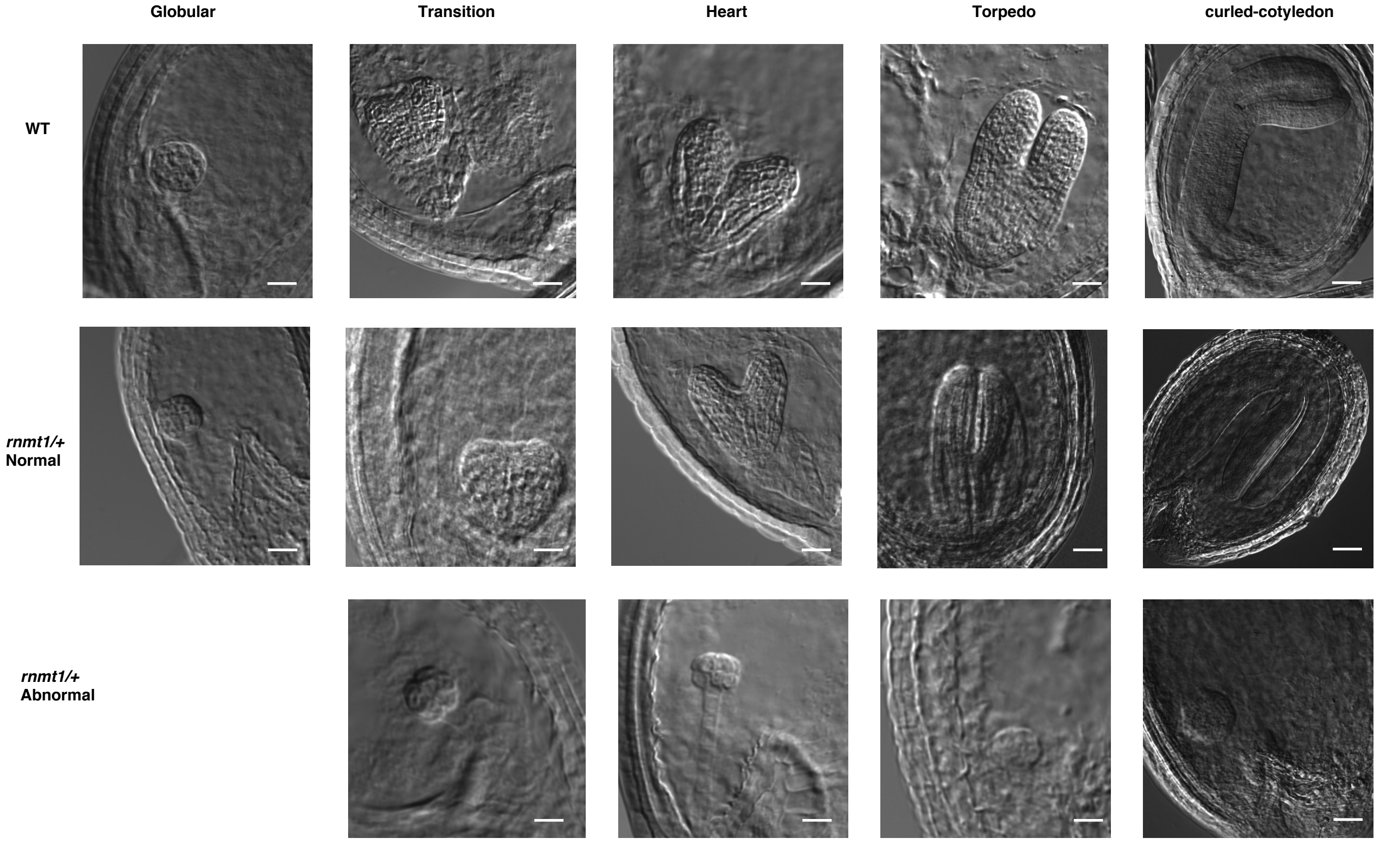

b

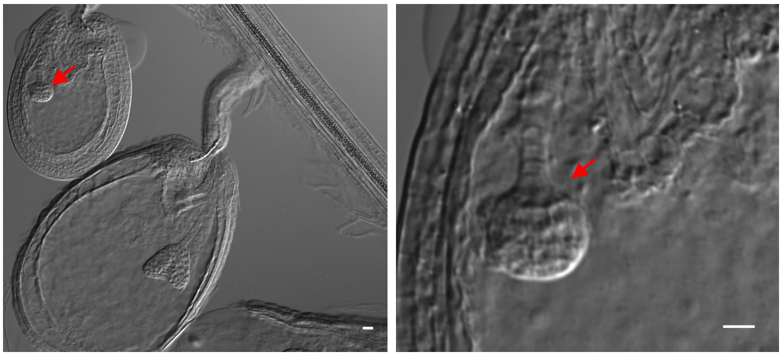

Supplementary Fig. 8. The *rnmt1/+* mutation causes arrest of embryo development at the globular stage. a, Developing embryos from WT and *rnmt1/+* plants at different stages of embryo development. Normal and abnormal embryos were from the same siliques. Some embryos in the heterozygous plants were arrested at the globular stage while the normal embryos proceeded to a later stage. **b**, Comparison of a normal embryo and an abnormal embryo (arrows) in the same silique from a heterozygous plant. The panel on the right is a close-up view of the abnormal embryo. The experiments in **a-b** were independently performed three times with similar results. Bars: 20 µm.

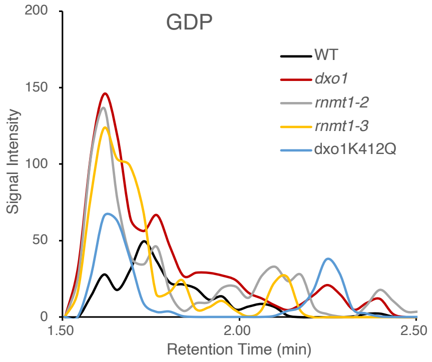

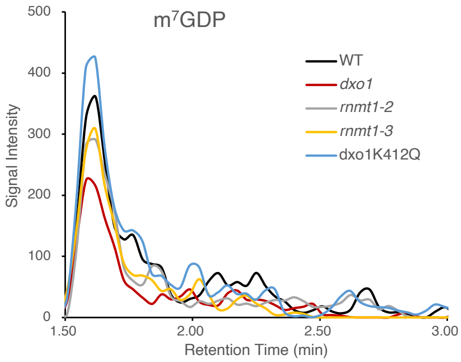

Supplementary Fig. 9. LC-MS peak plots of GDP and m7GDP corresponding to the data shown in Fig. 5. The X axis indicates retention time and Y axis indicates signal intensity. Three biological replicates were included in the analysis with similar results, and the result of one of the three replicates was shown in this figure.

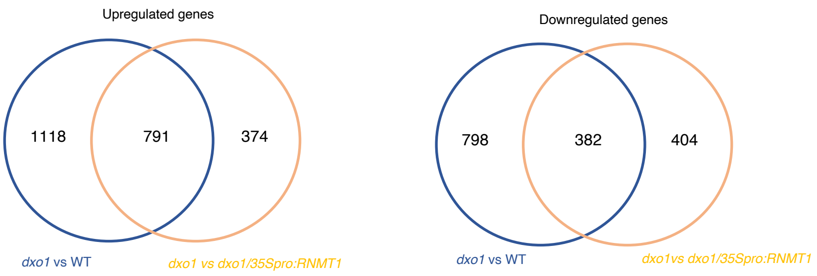

Supplementary Fig. 10. Overexpression of RNMT1 in *dxo1* partially complement alteration of gene expression caused by *dxo1*. Venn diagrams show overlaps of DEGs in *dxo1* compared to WT and *dxo1/35Spro:RNMT1.*

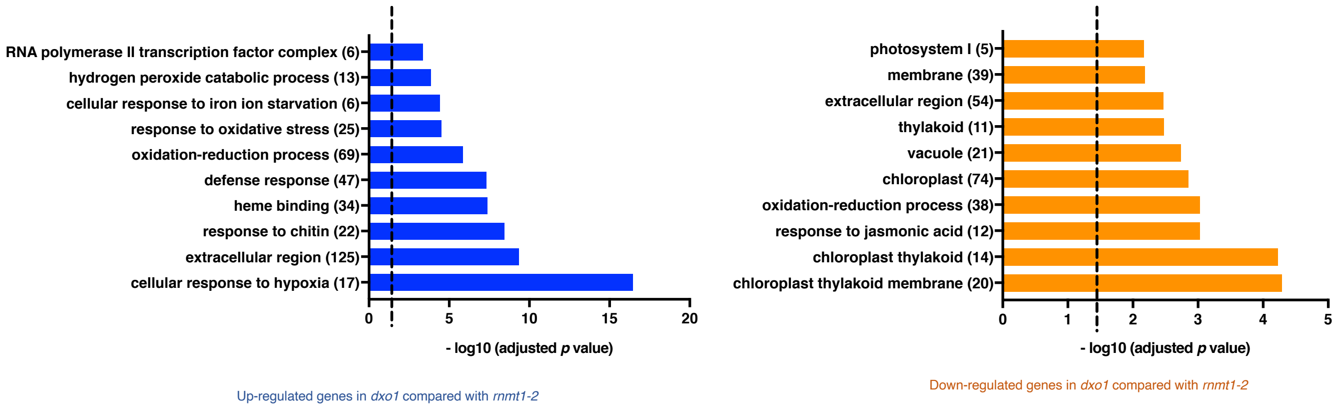

Supplementary Fig. 11. Top ten GO terms of up- and down-regulated DGEs in *dxo1* compared to *rnmt1*. The dotted line indicates the location of the FDR- adjusted *p* value = 0.05.

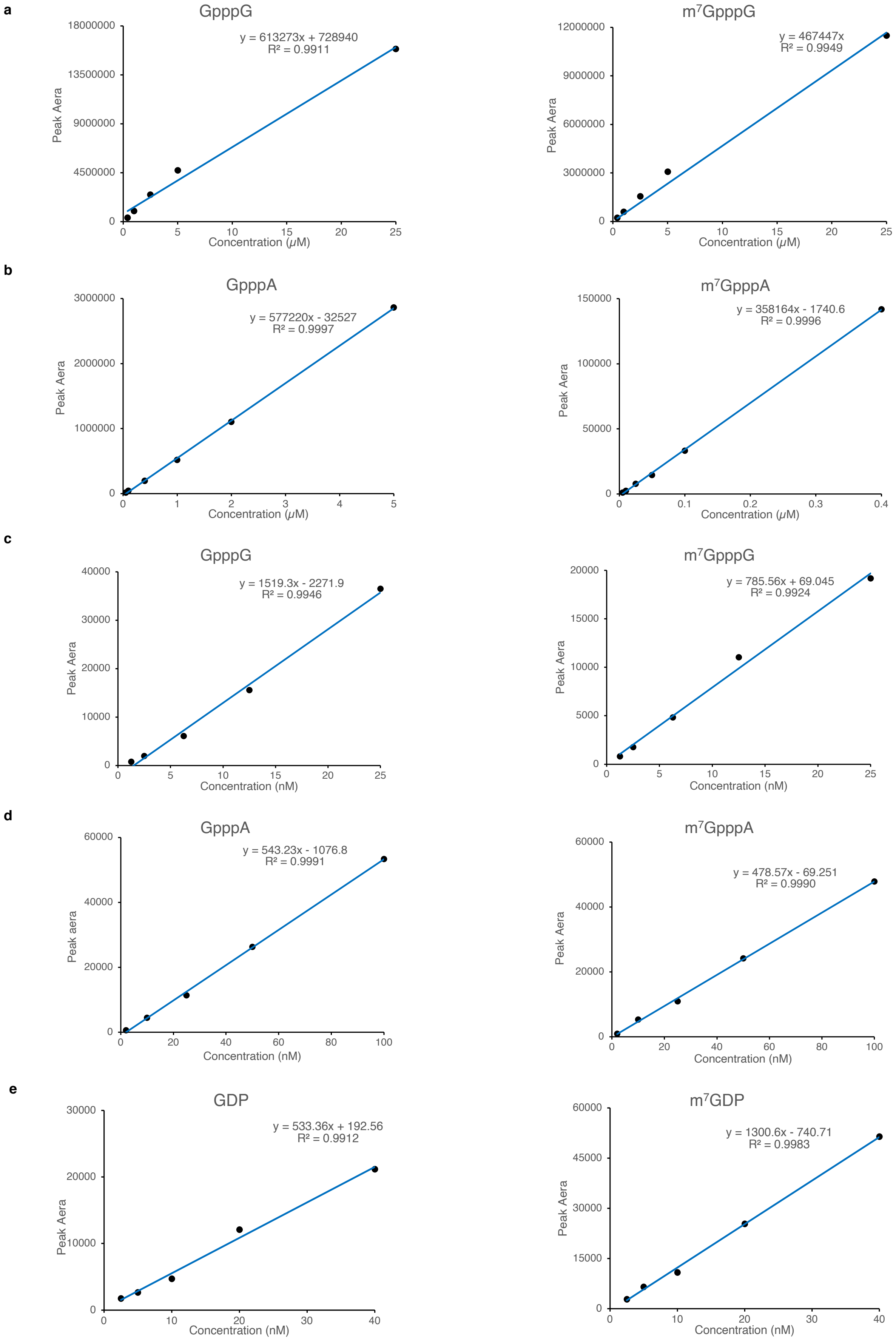

Supplementary Fig.12. Standard curves for GpppG/A, m^{7}GpppG/A, GDP and m^{7}GDP. a and **b**, standard curves for cap analogs corresponding to Fig. 3a and 3b. **c** and **d**, standard curves for RNAs corresponding to Fig. 3c and 3d. **e**, standard curves of GDP and m^{7}GDP corresponding to Fig. 5a. The X axis indicates concentration of standard samples and Y axis indicates peak area that is calculated according to signal intensity .

Supplementary Table 1. Chi-square test of segregation ratios from reciprocal crosses of *rnmt1/+* and WT

	rnmt1/+	*+/+*	χ^2 value	Fits
♀ WT x ♂ *rnmt1/+*	23	25	0.08	1:1
♀ *rnmt1/+* x ♂ WT	24	26	0.08	1:1

Chi-square test χ^2 0:05 = 3:84 (one degree of freedom).

Supplementary Table 2. Oligonucleotide primers in this study

Name	Sequence(5' to 3')	Application
DXO1-735-F	TATGCGTCGACATATGAGCTCATGGATTCACCGCCGAAGAAAAA	BiFC assay
DXO1-735-R	CCAGATCTGACTAGTGAGCTCTTACTGAGGTGGTGGTGGTGG	BiFC assay
RNMT1-736-F	GAAAAGTCGACATATGAGCTCATGAAACGAGGATTCTCCGATTCT	BiFC assay
RNMT1-736-R	CCAGATCTGACTAGTGAGCTCTCAGCTATCGATATACAAAACATCATCT	BiFC assay
RNMT1-HA-F	CTCCCCTTGCTCCGTGGATCCATGAAACGAGGATTCTCCGATTCT	Co-IP assay
RNMT1-HA-R	AACGTCGTATGGGTAAGGCCTGCTATCGATATACAAAACATCATCTTTCG	Co-IP assay
FCA-HA-F	CTCCCCTTGCTCCGTGGATCCATGAATGGTCCCCCAGATAGAGTAG	Co-IP assay
FCA-HA-R	AACGTCGTATGGGTAAGGCCTAGCTTTATTCTTCCACATGAGTTCT	Co-IP assay
rnmt1-mutant-F	CACCTTTCCTGCTTCGTCGG	Crispr lines validation
rnmt1-mutant-R	ACAGAGACTCTAAGAGTAAAAGAAACACTC	Crispr lines validation
1305-35SDXO1-F	CGGGGATCCTCTAGAGTCGACATGGATTCACCGCCGAAGAAAA	Overexpression plants
1305-35SDXO1-R	ATGGTCTTTGTAGTCAAGCTTCTGAGGTGGTGGTGGTGGTGAT	Overexpression plants
RNMT1-overexpr-F	AGTGGTACCATGAAACGAGGATTCTCCGATTC	Overexpression plants
RNMT1-overexpr-R	CTAAAGCTTGCTATCGATATACAAAACATCATC	Overexpression plants
DXO1-qPCR-F	GCTTCTTCCTCGGCCTGGAC	qPCR
DXO1-qPCR-R	GCTCACATGGCTCCTGAAAGT	qPCR
RNMT1-qPCR-F	AGGTGTCTTTATCGGAACAATGCCA	qPCR
RNMT1-qPCR-R	CAACAGCATCCTCGAGATGAAAC	qPCR
UBQ5-F	GACGCTTCATCTCGTCC	qPCR
UBQ5-R	GTAAACGTAGGTGAGTCC	qPCR
ACTIN2-F	GGTAACATTGTGCTCAGTGGTGG	qPCR
ACTIN2-R	AACGACCTTAATCTTCATGCTGC	qPCR
DXO1-28a-F	CAGCAAATGGGTCGCGGATCCATGGATTCACCGCCGAAGAA	recombinant protein expression
DXO1-28a-R	CTCGAGTGCGGCCGCAAGCTTTTACTGAGGTGGTGGTGGTGGTGA	recombinant protein expression
nDXO1-28a-R	CTCGAGTGCGGCCGCAAGCTTCTCAGAAACAAAACGTTCATCACGCT	recombinant protein expression
cDXO1-28a-F	CAGCAAATGGGTCGCGGATCCCTTAAGCTTTCGAAAAGCAAAGAAACG	recombinant protein expression
DXO1 K412Q-F	GTTTTACGTAGAGTTGCAAACAACTCGTG	recombinant protein expression
DXO1 K412Q-R	GCAACTCTACGTAAAACCTTTTTCCCTT	recombinant protein expression
RNMT1-28a-F	CAGCAAATGGGTCGCGGATCCATGAAACGAGGATTCTCCGATTCT	recombinant protein expression
RNMT1-28a-R	CTCGAGTGCGGCCGCAAGCTTTCAGCTATCGATATACAAAACATCATCT	recombinant protein expression
RNMT1-pGEX-F	ATCTGGTTCCGCGTGGATCCATGAAACGAGGATTCTCCGATTCTC	recombinant protein expression
RNMT1-pGEX-R	TCGAGTCGACCCGGGAATTCTCAGCTATCGATATACAAAACATCATCT	recombinant protein expression
RNMT1-protoplast-F	CTATTTACAATTACAGTCGACATGAAACGAGGATTCTCCG	Subcellular localization
RNMT1-protoplast-R	GCCCTTGCTCACCATGTCGACTCAGCTATCGATATACAA	Subcellular localization
DXO1-1-LP	GACTGAACTCAAACTCGGTCG	T-DNA insertion line
DXO1-1-RP	TGGGAAACAAGAACAAAGTGG	T-DNA insertion line
RNMT1-LP	TGTCTTTATCGGAACAATGCC	T-DNA insertion line
RNMT1-RP	TCATTTTCCCATTCTTCCTCC	T-DNA insertion line
c'RNMT1-AD-F	CTGGAATTCGATGCCAATGTTATTATAAAAAAACTCAGAG	yeast two-hybrid
c'RNMT1-AD-R	CTGGGATCCTCAGCTATCGATATACAAAACATCATCT	yeast two-hybrid
cRNMT1-AD-F	GCCATGGAGGCCAGTGAATTCTGCCAGTTTGCTATGCATTACTCA	yeast two-hybrid
cRNMT1-AD-R	CAGCTCGAGCTCGATGGATCCTCAGCTATCGATATACAAAACATCATCTT	yeast two-hybrid
mRNMT1-ADF	CTGGAATTCCTTTATGCTCGTCCTGACGATGCT	yeast two-hybrid
mRNMT1-AD-R	CTGGGATCCTGGCATTGTTCCGATAAAGACACCT	yeast two-hybrid
n'RNMT1-AD-F	CTGGAATTCATGAAACGAGGATTCTCCGATTCTC	yeast two-hybrid
nRNMT1-AD-F	GCCATGGAGGCCAGTGAATTCATGAAACGAGGATTCTCCGATTCT	yeast two-hybrid
n'RNMT1-AD-R	CTGGGATCCAGCATCGTCAGGACGAGCATAAAG	yeast two-hybrid
nRNMT1-AD-R	CAGCTCGAGCTCGATGGATCCTCAACTGCAGATATCAAAAGGGGCATC	yeast two-hybrid
DXO1-BD-F	ATGGCCATGGAGGCCGAATTCATGGATTCACCGCCGAAGAAAAA	yeast two-hybrid
DXO1-BD-R	CCGCTGCAGGTCGACGGATCCCTTACTGAGGTGGTGGTGGTGG	yeast two-hybrid
nDXO1-R	CAGCTCGAGCTCGATGGATCCTCACTCAGAAACAAAACGTTCATCACGC	yeast two-hybrid
cDXO1-F	ATGGCCATGGAGGCCGAATTCCTTAAGCTTTCGAAAAGCAAAGAAACG	yeast two-hybrid
