# Supplementary material for: Arabidopsis DXO1 activates RNMT1 to methylate the mRNA guanosine cap
Source: Nat Commun. 2023 Jan 13;14:202. doi: 10.1038/s41467-023-35903-8 (PMC9839713; doi:10.1038/s41467-023-35903-8)
Supplement: Supplementary file 2 — Description of Additional Supplementary Files [file 41467_2023_35903_MOESM2_ESM.pdf]

Description of Additional Supplementary Files:

Supplementary Data 1: Differentially expressed genes (DEGs)

Supplementary Data 2: GO term enrichment analysis of DEGs
